# The coadaptation theory for genomic imprinting

**DOI:** 10.1002/evl3.5

**Published:** 2017-05-03

**Authors:** Eleanor K. O'Brien, Jason B. Wolf

**Affiliations:** ^1^ Milner Centre for Evolution and Department of Biology & Biochemistry University of Bath Claverton Down Bath BA2 7AY United Kingdom

**Keywords:** Adaptive coordination, genomic imprinting, kin selection, social effects

## Abstract

Imprinted genes are peculiar in that expression of the two copies differs depending on whether the copy was maternally or paternally inherited. The discovery of this striking pattern of gene expression inspired myriad evolutionary theories, the most successful of which identify scenarios that create an asymmetry between the maternally and paternally inherited gene copies that favors silencing of one of the copies. Most notably, imprinting can evolve when gene dosage affects kin interactions (typically involving conflict) or when silencing enhances coadaptation by coordinating traits expressed by interacting kin. Although we have a well‐established theory for the former process (the “Kinship Theory”), the coadaptation process has only been explored for the specific case of interactions between mothers and offspring. Here, we fill this critical gap in our understanding by developing a general “Coadaptation Theory” that explains how imprinting can evolve to coordinate interactions between all types of relatives. Using a simple model in which fitness of an individual is determined by an interaction between its own phenotype (and hence genotype) and that of its social partner(s), we find that when the relatedness of interactants differs through their maternally versus paternally inherited gene copies, then selection favors expression of the allele through which relatedness is higher. The predictions of this Coadaptation Theory potentially apply whenever a gene underlies traits that mediate the outcome of conspecific interactions, regardless of their mechanism or the type of organism, and therefore provide a potential explanation for enigmatic patterns of imprinting, including those underlying adult traits. By providing simple testable predictions that often directly contrast with those derived from alternative theories, our model should play an important role in consolidating our understanding of the evolution of imprinting across genes and species, which will ultimately provide crucial insights into imprinted gene function and dysfunction.

**Impact Summary**

For most genes, no distinction is made between the copy inherited from mothers versus fathers, but for a peculiar subset of genes that show “genomic imprinting,” expression of each gene copy depends on its parental origin. Why did such an odd pattern of gene expression evolve? We address this fundamental question using a simple model where an individual's success in social interactions with relatives depends on the combination of traits that they and their social partners express. This “Coadaptation Theory” demonstrates that genomic imprinting can evolve because it leads to more successful social interactions by coordinating the traits expressed by interacting individuals. More specifically, imprinting benefits an individual because it enhances the compatibility between the gene copy that they express and the gene copy (or copies) expressed by their social partner(s). Understanding the conditions that favor genomic imprinting is important because it provides critical insights into the evolutionary processes shaping a key epigenetic feature of genomes. Such insights are broadly important because theories for the evolution of genomic imprinting have been used as central organizing principles for understanding the nature of imprinted genes across essentially all research areas. Thus, in addition to the value to evolutionary biologists, the Coadaptation Theory has potential utility for myriad problems in biology. For example, evolutionary theories for genomic imprinting have provided critical insights into the properties of imprinted genes in areas such as developmental biology by providing an explanation for the types of traits that they affect and the pathological consequences of loss of function mutations or epigenetic changes (such as changes that lead to a loss of imprinting at a gene). The Coadaptation Theory provides a predictive, testable framework that can be applied in such scenarios to understand why certain genes are imprinted, and more broadly, the utility of this critical genomic feature.

## Introduction

Genomic imprinting is an epigenetic phenomenon where the maternally and paternally inherited copies of a gene (hereafter matrigenic and patrigenic (Patten et al. 2014)) differ in their expression (Reik and Walter 2001). Imprinting evolved independently in mammals and angiosperms (Pires and Grossniklaus 2014), and may also occur in other taxa (Kronforst et al. 2008). Although a relatively small number of imprinted genes are known in mammals (∼100; Kelsey and Bartolomei 2012; Babak et al. 2015), they play a key role in many biological processes (Barlow and Bartolomei 2014). The peculiar characteristics of imprinted genes provoked a multitude of evolutionary explanations for its origin and distribution across genes and species (Patten et al. 2014; Spencer and Clark 2014). These models share a common feature that imprinting evolves when selection favors different expression of the two gene copies (Patten et al. 2014). Although unified by this essential property, models differ in assumptions underlying the processes that generate the differential selective pressures on the two gene copies. The predominant Kinship Theory (Haig 2002; Brandvain et al. 2011) postulates that imprinting is driven by conflict between the matrigenic and patrigenic copies over the level of gene expression that maximizes their “inclusive fitness” (which weighs the influence of the expression level on an individual's fitness and that of its relatives), with imprinting evolving to silence the copy with the lower expression optimum. However, although many interactions among relatives can be characterized by conflict, the outcome often depends on the combination of traits expressed by socially interacting individuals (Wolf 2000b). For example, cooperative interactions between individuals may be enhanced when interactants have compatible strategies, such as when there is some sort of “synergy” (Queller 1985, 2011; Corning and Szathmáry 2015). Such a pattern can favor adaptive coordination of traits expressed in interactants (Wolf and Brodie 1998). In the case where interactions are between a mother and her offspring, the Maternal‐Offspring Coadaptation theory has shown that imprinting can enhance such coordination (Wolf and Hager 2006) if it results in individuals only expressing the copy inherited from the care‐giving parent (typically the mother). This phenomenon is akin to the “greenbeard” effect, where fitness is enhanced through interactions with genetically similar individuals (Queller 2011; Haig 2014), and is modulated by imprinting because it increases the similarity of the allele expressed in offspring to those present in the care‐giving parent (Haig 1996; Wolf and Hager 2006; Haig 2014). This type of greenbeard effect includes the possibility that the locus mediates some sort of self‐recognition process (Haig 1996; Wolf and Hager 2009).

Both the Kinship and Maternal‐Offspring Coadaptation Theories were challenged by the discovery that many genes show imprinted expression after individuals are no longer receiving parental care (Wilkinson et al. 2007), including many expressed in the brain that affect social behavior (Davies et al. 2005; Wilkinson et al. 2007; Garfield et al. 2011), and several expressed in the mammary gland (Stringer et al. 2012, 2014; Cowley et al. 2014). The Kinship Theory has been generalized to include all interactions between relatives, with selection for imprinting resulting from relatedness asymmetries (such as those arising from sex‐biased dispersal or reproductive success) that generate conflict over the expression level favored by the matrigenic and patrigenic gene copies (Úbeda and Gardner 2010, 2011). In contrast, the Maternal‐Offspring Coadaptation Theory applies only to the limited case of interactions between mothers (or fathers) and their offspring, and even in that context imprinted expression in parents is not expected to evolve because both gene copies in a parent are equally related to their offspring (Wolf and Hager 2006). As with the Kinship Theory, the coadaptation process can presumably drive imprinting through other sorts of social interactions (Wolf et al. 2015), but this supposition remains unexplored. Here we formalize this conjecture by developing a general Coadaptation Theory that considers interactions between all sorts of relatives, to understand how selection for coadaptation in the social interactions of relatives can drive the evolution of imprinting.

## Methods

### THE MODEL

We develop a simple population genetic model to understand the basic conditions that favor imprinting for traits mediating social interactions. We present the fundamental logic and derivation of the model here and provide further details in the Supplementary Methods. A list of the model parameters are provided in Table 1.

**Table 1:** Definitions of terms and symbols used in coadaptation model of genomic imprinting, presented in the order in which they appear in the text

Parameter	Definition
*p*_1_	Frequency of the *A*_1_ allele
*p*_2_	Frequency of the *A*_2_ allele
*D*_*i*_	The phenotypic value of the direct trait for an individual with genotype *i*
*S*_*j*_	The phenotypic value of the social trait for an individual with genotype *j*
*i*	Index of focal individual's genotype at locus A (1 = *A*_1_ *A*_1_, 2 = *A*_1_ *A*_2_, 3 = *A*_2_ *A*_1_, 4 = *A*_2_ *A*_2_)
*j*	Index of social partner's genotype at locus A (1 = *A*_1_ *A*_1_, 2 = *A*_1_ *A*_2_, 3 = *A*_2_ *A*_1_, 4 = *A*_2_ *A*_2_)
*I*	Pattern of imprinting of the A locus for its effect on the direct trait, where −1 < *I* < 1, and positive values = matrigenic expression, negative values = patrigenic expression
*J*	Pattern of imprinting of the A locus for its effect on the social trait, where −1 < *J* < 1, and positive values = matrigenic expression, negative values = patrigenic expression
*a*_*d*_	Additive effect of the A locus on the direct trait
*a*_*s*_	Additive effect of the A locus on the social trait
*w*_*ij*_	Fitness of an individual with genotype *i* interacting with an individual with genotype *j*
ψ	Effect of the social interaction on fitness
*r*_*MM*_	Genetic identity coefficient (probability that gene copies are identical by descent) for the matrigenic gene copy in the focal individual and matrigenic gene copy in the social partner
*r*_*MP*_	Genetic identity coefficient for the matrigenic gene copy in the focal individual and patrigenic gene copy in the social partner
*r*_*PM*_	Genetic identity coefficient for the patrigenic gene copy in the focal individual and matrigenic gene copy in the social partner
*r*_*PP*_	Genetic identity coefficient for the patrigenic gene copy in the focal individual and patrigenic gene copy in the social partner
$\varphi_{GG^{\prime }}$	Genetic coefficient of kinship between focal individual and social partner (equal to average of the four relatedness terms above)
$\rho_{\mathrm{MM}}$	Expression‐weighted identity coefficient for the matrigenic gene copy in focal individual and the matrigenic gene copy in social partner
$\rho_{\mathrm{MP}}$	Expression‐weighted identity coefficient for the matrigenic gene copy in focal individual and the patrigenic gene copy in social partner
$\rho_{\mathrm{PM}}$	Expression‐weighted identity coefficient for the patrigenic gene copy in focal individual and the matrigenic gene copy in social partner
$\rho_{\mathrm{PP}}$	Expression‐weighted identity coefficient for the patrigenic gene copy in focal individual and patrigenic gene copy in social partner
$\varphi_{EE^{\prime }}$	Expression‐weighted coefficient of kinship between focal individual and social partner
*f_*ij*_*	Frequency of social interactions between focal individuals with genotype *i* and social partners with genotype *j*
$\bar{w}$	Population mean fitness
*δ*	Effect of modifier allele *B*_1_ on imprinting of the A locus for the direct trait
*σ*	Effect of modifier allele *C*_1_ on imprinting of the A locus for the social trait
cov*_DS_*	Covariance of the direct and social traits expressed by interactants
*β_I_*	Selection gradient favoring imprinting of the effect of the A locus on the direct trait
*β_J_*	Selection gradient favoring imprinting of the effect of the A locus on the social trait
θ	Total fitness effect of the A locus, combining the effect of the locus on the direct and social traits (*a_d_*, *a_s_*) and the effect of social interactions on fitness ψ

#### Genetics of phenotypes and fitness

We consider a locus (the “A locus”) that has two alleles, *A*_1_ and *A*_2_, with frequencies *p*_1_ and *p*_2_, respectively (where *p*_1_ + *p*_2_ = 1). The four ordered diploid genotypes (*A*_1_ *A*_1_, *A*_1_ *A*_2_, *A*_2_ *A*_1_, and *A*_2_ *A*_2_, with the matrigenic copy given first) occur in Hardy–Weinberg proportions (i.e., frequencies are $p_{1}^{2}$, *p*_1_ *p*_2_, *p*_2_ *p*_1_, $p_{2}^{2}\;$, respectively).

We assume that the locus directly affects some trait possessed by “focal” individuals (the “direct trait,” with value *D*_*i*_) and also affects some trait expressed in the individuals with whom the focal individuals interact (the “social trait,” with value *S*_*j*_; where the subscripts index the four ordered genotypes, with 1 = *A*_1_ *A*_1_, 2 = *A*_1_ *A*_2_, 3 = *A*_2_ *A*_1_, 4 = *A*_2_ *A*_2_). From the perspective of the focal individuals, the social trait can be considered to be a component of the social environment they experience. The model applies equally to the case where there is one trait, such that the direct trait also influences the social environment (i.e., so *D*_*i*_ = *S*_*j*_ when *i* = *j*), or there are two distinct traits, one direct and one social (i.e., *D*_*i*_ ≠ *S*_*j*_ when *i* = *j*).

Imprinting modulates the influence of the locus on the traits by determining the expression of the two alleles within an individual genotype. The degree and pattern of imprinting are given by the parameters *I* (‒1 ≤ *I* ≤ 1) for the direct trait and *J* (‒1 ≤ *J* ≤ 1) for the social trait. Positive values of *I* and *J* indicate expression biased toward the matrigenic copy (i.e., some degree of silencing of the patrigenic copy) and negative values indicate a bias toward the patrigenic copy for the relevant trait. When *I* or *J* = 0 there is normal biallelic expression and when |*I*| or |*J*|= 1 one copy is silenced, resulting in uniparental expression. This model structure allows the locus to potentially show different patterns of expression for the different traits (or for the same trait at different times in life), which is consistent with the empirical observation that imprinted genes can show different expression patterns in different contexts (tissues, timings, etc.; Baran et al. 2015), including cases where the same gene can show opposite expression patterns (i.e., maternal vs. paternal) in different tissues (e.g., *Grb10*; Garfield et al. 2011)).

**Figure 1:**
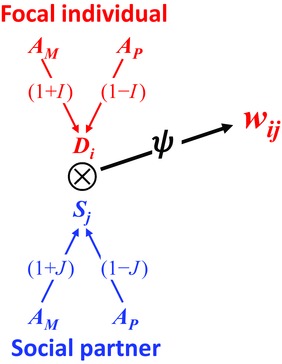
A diagrammatic representation of the model for trait genetics and fitness. The gene copies (*A*, with subscripts *M* and *P* that indicate the matrigenic and patrigenic copies, respectively) in the focal individual and their social partner affect expression of the direct (*D*_*i*_) and social (*S*_*j*_) traits in those individuals, respectively. The expression of the gene copies is weighted by terms that account for the pattern of imprinting, which appear overlaid on the arrow connecting the gene copy with the value of the trait. The social interaction (indicated by the circle with an X at the interface of the traits in the interacting individuals) has an effect (given by ψ) on the fitness of the focal individual (*w*_*ij*_). Components in red are properties of the focal individual and those in blue are properties of their social partner. The fitness effects are not necessarily reciprocal (e.g., the case of an interaction of an offspring and its mother), so a separate representation would be required for the fitness of the social partner.

To connect the allelic variation to trait expression, we build from the classic additive quantitative genetic model, where the two alleles in a genotype have independent effects on trait expression (Falconer and Mackay 1996). Under this general model, the influence of a diploid genotype on trait expression can be defined as the average phenotype associated with the component alleles, which allows for a simple means of incorporating imprinting by weighting this average by the degree of expression of the alleles. We assume that the *A*_1_ allele has an effect of +*a*_*d*_ on the direct trait and of +*a*_*s*_ on the social trait, while the *A*_2_ allele has effects of ‒*a*_*d*_ and ‒*a*_*s*_, respectively. In the absence of imprinting, the four ordered genotypes (listed in the order of *A*_1_ *A*_1_, *A*_1_ *A*_2_, *A*_2_ *A*_1_, and *A*_2_ *A*_2_) would therefore have the phenotypic values of *D*_*i*_ = [+*a*_*d*_, 0, 0, ‒*a*_*d*_] and *S*_*i*_ = [+*a*_*s*_, 0, 0, ‒*a*_*s*_], matching the pattern expected for the classic quantitative genetic model (Falconer and Mackay 1996). To calculate the expected phenotypic values with imprinting, we weigh the effect of an allele by the pattern of expression. For the matrigenic allele, the effect is weighted by (1 + *I*) for the direct trait and (1 + *J*) for the social trait, while the effect of the patrigenic copy is weighted by (1 – *I*) and (1 – *J*) for these traits, respectively (see Fig. 1). For example, the phenotypic value for the direct trait of the genotype *A*_1_ *A*_1_ is ½[(1 + *I*)*a*_*d*_ + (1 – *I*)*a*_*d*_], which simplifies to *a*_*d*_ for all patterns of imprinting, since the *A*_1_ allele is always the allele being expressed. Likewise, the phenotypic value of the direct trait associated with genotype *A*_1_ *A*_2_ is ½[(1 + *I*)*a*_*d*_ – (1 – *I*)*a*_*d*_], which simplifies to *Ia*_*d*_, while the direct trait phenotypic value associated with *A*_2_ *A*_1_ is ½[–(1 + *I*)*a*_*d*_ + (1 – *I*)*a*_*d*_], which simplifies to –*Ia*_*d*_. This difference between the reciprocal heterozygotes highlights the impact of imprinting on trait expression, where a heterozygote will have the phenotype associated with the matrigenic allele under maternal expression, but the patrigenic allele under paternal expression. Overall, the phenotypic values of the four genotypes at the A locus (again ordered as *A*_1_ *A*_1_, *A*_1_ *A*_2_, *A*_2_ *A*_1_, and *A*_2_ *A*_2_) for the direct trait are given by the vector **D_A_** = [*a*_*d*_, *Ia*_*d*_, ‒*Ia*_*d*_, ‒*a*_*d*_], and for the social trait by **S_A_** = [*a*_*s*_, *Ja*_s_, ‒*Ja*_s_, ‒*a*_*s*_].

The fitness of an individual with genotype *i* interacting with an individual of genotype *j* (*w*_*ij*_) is determined by an interaction between the phenotypic value of its own direct trait (*D*_*i*_) and the phenotypic value of the social trait of its social partner (*S*_*j*_). The effect of the interaction on the fitness of the focal individual is given by the coefficient ψ (see Fig. 1):

(1) $$w_{ij}=1+D_{i}S_{j}\psi$$

These fitness values defined by equation (1) are given by the vector wA=1+ψ vec (SADAT)(where the operation “vec” achieves vectorization).

#### Relatedness of interactants with imprinting

**Figure 2:**
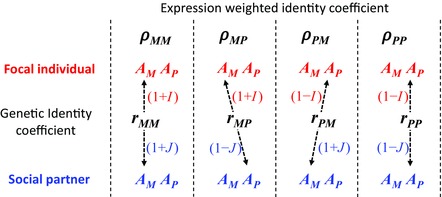
An illustration of the four expression weighted identity coefficients (ρ_*MM*_, ρ_*MP*_, ρ_*PM*_, and ρ_*PP*_). In each case, the parental origin of the gene copies (*A*) in the focal individual and their social partner are denoted as *M* and *P*, where the letter indicates the matrigenic and patrigenic copies, respectively. The relatedness of each pair of gene copies in the interactants appears overlaid on the line connecting that particular pair of copies. These are given by four genetic identity coefficients (*r*_*MM*_, *r*_*MP*_, *r*_*PM*_, and *r*_*PP*_) that indicate the probability that the particular alleles present at that pair of gene copies are identical by descent. Each of the patterns of genetic identity are modified by the pattern of imprinting, which is represented by weighting the relationship from the gene copies in the focal individual by (1 + *I*) for the matrigenic copy and (1 – *I*) for the patrigenic copy and from the copies in the social partner by (1 + *J*) for the matrigenic copy and (1 – *J*) for the patrigenic copy. Components in red are properties of the focal individual and those in blue are properties of their social partner.

We measure relatedness using four genetic identity coefficients, *r*_*MM*_, *r*_*MP*_, *r*_*PM*_, and *r*_*PP*_ (following Jacquard 1972), which give the pair‐wise probabilities that the matrigenic (*M*) or patrigenic (*P*) gene copies in the focal individual are identical by descent (IBD) with each copy in its social partner (where the first subscript indicates the parental origin of the gene copy in the focal individual and the second that of the partner). The offspring‐mother case provides a simple example: Offspring inherit their matrigenic gene copy from their mother, and there is an equal probability that the offspring inherits its mother's own matrigenic or patrigenic copy, making the genetic identity coefficients *r*_*MM*_ = *r*_*MP*_ = ½ (where the first subscript indicates the offspring's gene copy and the second the mother's). In a randomly mating population, offspring are not related to their mother through their patrigenic copy, therefore *r*_*PM*_ = *r*_*PP*_ = 0. The average of the four genetic identity coefficients gives the coefficient of kinship for the pair [$\phi_{GG^{\prime }}$ = ¼(*r*_*PM*_ + *r*_*PP*_ + *r*_*MM*_ + *r*_*MP*_)], which is half the coefficient of relatedness (Jacquard 1972). Thus, for offspring and their mothers, $\varphi_{GG^{\prime }}$ = ¼, giving the expected coefficient of relatedness of ½. However, when a locus is imprinted, fitness depends on the alleles that an individual and its social partners express, not their diploid genotypes. Hence, to have a relevant and functional measure of relatedness (Queller 2011) we need to weigh the measure of genetic identity by the pattern of expression of those copies to produce an expression weighted measure of relatedness (see Fig. 2). For example, consider relatedness of individuals through their matrigenic copies; the genetic identity of the gene copies is *r*_*MM*_, which is weighted by the expression of the matrigenic copy in focal individuals (1 + *I*) for the direct trait and by (1 + *J*) in social partners for the social trait, making the expression weighted identity coefficient for the matrigenic copies $\rho_{\mathrm{MM}}=\left(1+I\right)\left(1+J\right)r_{\mathrm{MM}}$. It follows that $\rho_{\mathrm{MP}}=\left(1+I\right)\left(1-J\right)r_{\mathrm{MP}}$, $\rho_{\mathrm{PM}}=\left(1-I\right)\left(1+J\right)r_{\mathrm{PM}}$, and $\rho_{\mathrm{PP}}=\left(1-I\right)\left(1-J\right)r_{\mathrm{PP}}$. The average of the four expression weighted identity coefficients gives the expression weighted coefficient of kinship of interactants:

(2) $$\begin{array}{ccc} \varphi_{EE^{\prime }} & = & \frac{1}{4}\left(\rho_{\mathrm{MM}}+\rho_{\mathrm{MP}}+\rho_{\mathrm{PM}}+\rho_{\mathrm{PP}}\right)\\ & = & \frac{1}{4}\left(\begin{array}{c} \left(1+I\right)\left(1+J\right)r_{\mathrm{MM}}\\ +\left(1+I\right)\left(1-J\right)r_{\mathrm{MP}}\\ +\left(1-I\right)\left(1+J\right)r_{\mathrm{PM}}\\ +\left(1-I\right)\left(1-J\right)r_{\mathrm{PP}} \end{array}\right) \end{array}$$

When there is no imprinting, this equation reduces to the coefficient of kinship ($\varphi_{GG^{\prime }}$), which averages the four types of relatedness. However, when there is imprinting, the expression weighted coefficient of kinship only averages over the gene copies being expressed. For example, if only the matrigenic copy is expressed for the direct and social traits (*I* = *J* = 1) then $\varphi_{EE^{\prime }}=r_{\mathrm{MM}}$, demonstrating that only matrigenic kinship is important. Likewise, if only the patrigenic copy is expressed for the direct trait but there is no imprinting for the social trait (*I* = −1, *J* = 0), then $\varphi_{EE^{\prime }}=\frac{1}{2}\left(r_{\mathrm{PM}}+r_{\mathrm{PP}}\right)$, so only the patrigenic copy in the focal individual matters when measuring kinship.

#### Frequencies of social interactions

The frequency of each of the possible pair‐wise allelic combinations in interactants is a function of the four relatedness terms (*r*_*MM*_, *r*_*MP*_, *r*_*PM*_, and *r*_*PP*_) and allele frequencies (Table S1). The frequencies of these pair‐wise allelic combinations determine the frequencies of interactions between each of the focal‐partner diploid genotype combinations, $f_{ij}$ (that make up the vector of frequencies for the A locus, **F_A_**), which are given in Table S2. These frequencies can be used along with the vector of fitness to calculate population mean fitness as: $\bar{w}=w_{A}\cdot F_{A}$.

#### Evolution of imprinting modifiers

To understand the conditions that favor the evolution of imprinting we consider selection on an allele at the B locus that modifies imprinting of the A locus for its effect on the direct trait and an allele at the C locus that modifies imprinting of the A locus for its effect on the social trait. Because the basic results of this analysis do not depend on the pattern of linkage disequilibrium between loci, we simplify the presentation here by assuming that the modifier locus is unlinked to the A locus. This approach provides a simple account of the conditions that favor the evolution of imprinting at the A locus with minimal loss of generality. Because of the complexity of the multilocus analysis, we provide a brief description here and additional details in the Supplementary Methods. To further simplify the presentation we consider evolution of each modifier locus individually since modelling their simultaneous evolutionary dynamics does not alter the nature of the results, but adds considerable complexity to the presentation.

The B locus has two alleles, *B*_1_ and *B*_2_, with frequencies *x*_1_ and *x*_2_. Genotypes occur in Hardy–Weinberg proportions, so genotype frequencies follow the A locus presentation. The frequencies of interactions between individuals with the various B locus genotypes follow the same patterns as for the A locus, and because relatedness affects allele sharing at all loci in the genome in the same way, the identity coefficients among interactants are also the same (see Tables S3a and S4a). We assume that the *B*_1_ allele causes some degree of imprinting (denoted *δ*) of the A locus for its effect on the direct trait while the *B*_2_ allele does not. Therefore, it is the B locus genotype that determines the value of the imprinting parameter *I* for a given genotype (and hence has the same consequences for trait expression and fitness). The overall influence of a given B locus genotype on imprinting of the A locus for its effect on the direct trait is determined by the average influence of the pair of alleles, so the imprinting effects of the four B locus genotypes (listed as *B*_1_ *B*_1_, *B*_1_ *B*_2_, *B*_2_ *B*_1_, and *B*_2_ *B*_2_) on expression of the A locus for the direct trait are *δ*, ½*δ*, ½*δ*, and 0. As with the imprinting parameter *I*, −1≤ *δ* ≤ +1 with positive values indicating expression biased toward the matrigenic copy (i.e., some degree of silencing of the patrigenic copy) and vice versa. When the imprinting modifier allele is segregating at the B locus, the population imprinting parameter *I* corresponds to the average degree of imprinting of the A locus for its effect on the direct trait (which depends on the frequency of the *B*_1_ allele), such that *I* would simply equal *x*_1_. Further details of the properties of the A and B two‐locus system (phenotypes and genotype frequencies) are given in the Supplementary Methods.

Evolution of imprinting for the effect of the A locus on the direct trait is determined by the change in the frequency of the imprinting modifier (*B*_1_) allele, $\Delta x_{1}$. This change is determined by the covariance between the proportion of an individual's B locus alleles that are *B*_1_ with its relative fitness ($w_{ij}/\bar{w}$) (Price 1970): $\mathrm{cov}\left(B_{1},w_{ij}\right)\bar{w}=\Delta x_{1}\bar{w}$ (see Supplementary Methods).

The basic properties of the C locus follow those of the B locus except that it modifies imprinting of the A locus for its effect on the social trait. The two alleles, *C*_1_ and *C*_2_ have frequencies *y*_1_ and *y*_2_, with the *C*_1_ allele causing a degree of imprinting of the A locus for its effect on the social trait, which is given by the parameter *σ*. The pattern of imprinting of the A locus for the social trait associated with the four C locus genotypes (listed as *C*_1_ *C*_1_, *C*_1_ *C*_2_, *C*_2_ *C*_1_, and *C*_2_ *C*_2_) are *σ*, ½*σ*, ½*σ*, and 0. These define the value of the imprinting parameter *J* for each C locus genotype (with the properties of *σ* following that of *J*), and hence have the same consequences for trait expression and fitness. Note that when the imprinting modifier allele is segregating at the C locus, the imprinting parameter *J* corresponds to the average degree of imprinting for the effect of the A locus on the social trait (which depends on the frequency of the *C*_1_ allele), such that *J* would simply equal *y*_1_.

As with the B locus, evolution of imprinting of the A locus for its effect on the social trait is determined by the change in the frequency of the imprinting modifier (*C*_1_) allele, $\Delta y_{1}$. This change is determined by the covariance between the proportion of an individual's C locus alleles that are *C*_1_ with its relative fitness ($w_{ij}/\bar{w}$) (Price 1970): $\mathrm{cov}\left(C_{1},w_{ij}\right)\bar{w}=\Delta y_{1}\bar{w}$ (see Supplementary Methods).

## Results and Discussion

We model a simple scenario where an individual's fitness depends on an interaction between (i.e., the combination of) the value of a trait they express (the phenotypic value of their “direct trait,” *D*_*i*_) and the value of a trait expressed by their social partner(s) (the phenotypic value of their partner's “social trait,” *S*_*j*_) (see equation 1). The direct and social traits can potentially be completely different traits linked by pleiotropy (e.g., resource provisioning by mothers and resource demand by offspring; [Cowley et al. 2014]) or can reflect the direct and social effects arising from the same underlying trait (e.g., aggressive behavior in competing individuals; [Wilson et al. 2009]). This scenario is analogous to the phenomenon of synergy in a social interaction, where fitness depends on the combination of traits of interactants and fitness is highest when combinations match or are otherwise compatible (Queller 1984, 2011; Corning and Szathmáry 2015). We model the genetic basis of these traits by building on the classic additive quantitative genetic model where a locus has an additive effect on the expression of these two traits. From this genetic perspective, the pattern of selection arising from social interactions can also be conceptualized as a “genotype‐by‐social‐environment interaction” or likewise as a type of epistatic interaction between the genotypes of interactants (Haig 1996; Wolf 2000b). These sorts of interaction effects have been documented in a wide array of systems across a diversity of social relations (Wolf et al. 2014), including those between parents and offspring (Haig 1996; Wolf 2000a).

When the fitness effect of social interactions depends on the combination of traits that individuals express (as in our model), individuals will have higher fitness when they experience “compatible” social environments (i.e., social partners with trait values compatible with their own phenotype). Consequently, selection is expected to favor mechanisms that lead to adaptive coordination between traits expressed by interactants (Corning and Szathmáry 2015). Such “social coadaptation” can arise from several sources (Wolf et al. 2014), most notably from relatedness, where common ancestry creates an association between the genotypes (and hence phenotypes) of interactants. However, because the outcome of the social interaction depends on the combination of traits (rather than genotypes) expressed by interactants, the contribution of relatedness to social coadaptation necessarily depends on the pattern of gene expression. We capture this phenomenon using an expression weighted coefficient of kinship, $\phi_{EE^{\prime }}$, which weighs the probability that individuals share alleles that are identical by descent (IBD) by their expression pattern. For example, if individuals share alleles that are IBD but at least one of those alleles is not expressed due to imprinting, then that genetic relationship would not contribute to the overall weighted coefficient of kinship, which is logical since that component of relatedness has no fitness consequences.

The overall pattern of social coadaptation between the traits expressed by interactants can be captured by the phenotypic covariance of the traits they express in the interaction (cov_*DS*_, where *D*_*i*_ is expressed in a focal individual and *S*_*j*_ in their social partner[s]):

(3a) co vDS=2p1p2adasφEE′

The RHS of this equation has two components, the additive genetic covariance between the direct and social traits ($2p_{1}p_{2}a_{d}a_{s}$) and the expression weighted coefficient of kinship ($\varphi_{EE^{\prime }}$) of interactants (see equation 2). To elucidate the role of imprinting, we can rewrite this equation (3a) in an expanded form:

(3b) co vDS=12p1p2adas1+I1+JrMM+1+I1−JrMP+1−I1+JrPM+1−I1−JrPP

which demonstrates that when imprinting leads to the expression of the gene copies through which interactants are related and silencing of those through which they are not, it enhances the contribution of relatedness to the social covariance. For example, if interactants are only related through their matrigenic copies (i.e., *r*_*MM*_ > 0) then maternal expression for the direct and social traits (i.e., *I* = *J* = 1) increases the contribution of relatedness to the social covariance by a factor of four (i.e.,  co vDS=2p1p2adasrMM with maternal expression compared to  co vDS=12p1p2adasrMM with normal expression). Hence, such a pattern can be said to “coordinate” the interaction by enhancing the compatibility of the gene copies that interactants express.

The importance of social coadaptation for the expected fitness of individuals is captured by the expression for population mean fitness (see Methods):

(4) w¯=ψadasp2−p12+ co vDS

The first term inside the brackets simply reflects expected fitness under random interactions, which is not influenced by imprinting. Hence, the only term in this equation that is affected by imprinting or relatedness is the social covariance (and so imprinting only affects expected fitness through its influence on the social covariance, and its influence is necessarily mediated by relatedness, eq. 3b).

The critical role that the phenotypic relationship between interactants (i.e., the social covariance) plays in the expected fitness of individuals in social interactions (i.e., population mean fitness, eq. 4) is conceptually analogous to the role it plays in “kind selection” (Queller 2011). Kind selection occurs when the fitness effect of social interactions is entirely mediated by the combination of traits expressed by interactants (not their genotypes), and hence differs from kin selection because the critical factor is “trait identity,” not genetic identity (where trait identity is a measure of the match between the traits of interactants). From this perspective, we can see that imprinting is important because it modulates the contribution of genetic identity to trait identity. The type of trait mediated fitness effects of social interactions in kind selection (and hence in our model) is logically analogous to those of the “greenbeard” concept, where phenotypic matching facilitates positive social interactions, and hence our model can be viewed as a type of greenbeard model (Haig 2013).

We can formally examine the conditions under which imprinting is favored by considering the conditions under which selection favors some modifier that changes the imprinting status of a locus affecting traits that mediate social interactions. The pattern of selection is captured by selection gradients on modifier alleles at loci where one allele causes imprinting and one does not (see Methods and Supplementary Methods). These selection gradients give both the conditions for invasion by the modifier and the fixation conditions. For the direct trait, the selection gradient (*β*_*I*_) on a modifier allele with a given pattern of imprinting is:

(5a) $$\beta_{I}\;=\;p_{1}p_{2}a_{d}a_{s}\psi \;\left[\left(1\;+\;J\right)\;\left(r_{\mathrm{MM}}\;-\;r_{\mathrm{PM}}\right)+\left(1\;-\;J\right)\;\left(r_{\mathrm{MP}}-r_{\mathrm{PP}}\right)\right]$$

Positive gradients indicate conditions that would favor a modifier causing maternal expression of the A locus for the direct trait (i.e., selection favors a modifier allele causing a positive value of *δ*) while negative values would favor a modifier causing paternal expression (i.e., selection favors a modifier allele causing a negative value of *δ*). The selection gradient can be simplified by combining the effect parameters (ad,as and ψ) that mediate each of the steps in the connection between allelic variation at the locus and fitness, into a single selection parameter, θ. This selection parameter can be viewed as a sort of “synergism” (Queller 2011) or “social epistasis” (Wolf et al. 2014) coefficient that reflects the overall influence of interactions between genotypes on the fitness of a focal individual. Substituting this coefficient into equation (5a) yields:

(5b) $$\beta_{I}\;=\;\theta p_{1}p_{2}\left[\left(1\;+\;J\right)\left(r_{\mathrm{MM}}\;-\;r_{\mathrm{PM}}\right)\;+\;\left(1\;-\;J\right)\left(r_{\mathrm{MP}}\;-\;r_{\mathrm{PP}}\right)\right]$$

which demonstrates that selection favoring imprinting is a product of three components: the total fitness effect of the locus through social interactions (θ), the amount of allelic variation at the locus ($p_{1}p_{2}$), and a set of expression weighted relatedness differentials (the terms in brackets). Importantly, this selection gradient not only describes the conditions favoring an imprinting modifier, but more generally describes the conditions under which selection favors imprinting for the direct trait, regardless of the mechanism (e.g., whether control of imprinting is *cis* or *trans*). This can be demonstrated by partial differentiation of population mean fitness (eq. 4) with respect to imprinting of the A locus for the direct trait, $\partial \bar{w}/\partial I$, which produces the same basic form as equation (5a). This means that the expected fitness of an individual is higher if they have an appropriate pattern of imprinting at the A locus (i.e., whichever pattern is favored), and hence imprinting can be considered to be directly adaptive. Furthermore, because imprinting only affects expected (mean) fitness through its influence on the social covariance (eq. 4), selection favoring imprinting must do so because of the effect it has on the social covariance (meaning that the selection gradient, therefore, also reflects the influence of imprinting on the social covariance).

For the social trait, the selection gradient (*β*_*J*_) on a modifier causing a given pattern of imprinting of the A locus is:

(6) $$\beta_{J}\;=\;\varphi_{GG^{\prime }}p_{1}p_{2}\theta \left[\left(1+I\right)\;\left(r_{\mathrm{MM}}-r_{\mathrm{MP}}\right)+\left(1-I\right)\;\left(r_{\mathrm{PM}}-r_{\mathrm{PP}}\right)\right]$$

As with the selection gradient on a modifier causing imprinting of the A locus for its effect on the direct trait (eq. 5), positive gradients indicate conditions that favor a modifier causing maternal expression of the A locus for its effect on the social trait (i.e., selection favors a modifier allele causing a positive value of *σ*) while negative values favor a modifier causing paternal expression (i.e., selection favors a modifier allele causing a negative value of *σ*). The basic form of this selection gradient matches that of the direct effect (eqs. 5a and 5b), except it includes the genetic coefficient of kinship of the interactants ($\varphi_{GG^{\prime }}$). Relatedness of interactants is critically important for the evolution of imprinting of the effect of the A locus on the social trait because the value of the social trait expressed by an individual influences the fitness of relatives, not their own fitness. Consequently, imprinting of the A locus for its effect on the social trait evolves by kin selection (Haig 2002). As discussed above for the direct trait, the conditions under which kin selection favors a modifier of imprinting of the effect of the A locus on the social trait also give the general conditions under which imprinting is favored, regardless of the mechanism. This can be demonstrated by partial differentiation of population mean fitness (eq. 4) with respect to imprinting of the A locus for the social trait (*J*), $\partial \bar{w}/\partial J$, which has the same basic form as equation (6) except it needs to be weighted by the genetic coefficient of kinship to translate the relationship into kin selection. As with imprinting for the direct trait, kin selection favors imprinting of the effect of the A locus on the social trait because of its influence on the social covariance.

Given a particular fitness effect of the locus (θ), the patterns of imprinting that are favored for the direct and social traits depends on a set of relatedness asymmetries (eqs. 5b and 6). For example, in the gradient favoring imprinting of the direct effect (eq. 5b), the asymmetry has two components, $\left(1+J\right)\left(r_{\mathrm{MM}}-r_{\mathrm{PM}}\right)$ and $\left(1-J\right)\left(r_{\mathrm{MP}}-r_{\mathrm{PP}}\right)$. The first of these terms gives the difference in the relatedness of the focal individual's matrigenic ($r_{\mathrm{MM}}$) and patrigenic ($r_{\mathrm{PM}}$) copies to the matrigenic copy of their partner, while the second term gives the difference in relatedness of these copies to the partner's patrigenic copy ($r_{\mathrm{MP}}$ and $r_{\mathrm{PP}}$). Maternal expression of the locus for its effect on the direct trait is favored when the matrigenic copies in focal individuals are more related to the alleles in their social partners than are the patrigenic copies ($r_{\mathrm{MM}}>r_{\mathrm{PM}}$ and $r_{\mathrm{MP}}>r_{\mathrm{PP}}$) and vice versa. However, these terms have to be weighted by the pattern of imprinting for the effect of the locus on the social trait because this determines which copy in the partners is actually expressed (and hence which copy matters). If only the matrigenic copy is expressed for the social trait (*J* = 1), then logically only the matrigenic copy in social partners matters (since the patrigenic copy would be silenced) and hence only the first of the relatedness differentials would matter. In general, these asymmetries demonstrate that selection favors expression of the gene copy that has higher relatedness with the relevant copy in their social partner(s) (which is perhaps most likely when one type of relatedness is zero).

The selection gradients (eqs. 5 and 6) clearly demonstrate that the strength of selection favoring imprinting of the locus for its effect on the direct or social trait through coadaptation depends on the level of genetic variation at the locus present in the population (i.e., the value of *p*_1_ *p*_2_ in eqs. 5 and 6). It is, therefore, important to keep in mind that the presence of variation is necessarily a key factor in driving the coadaptation process (Haig 2014; Wilkins 2014; Ubeda and Gardner 2015), and hence processes that generate or maintain variation are likely to be important determinants of the types of traits and scenarios where coadaptation will be important. Our model makes no assumptions about the processes that introduce or maintain this variation (and it is outside the scope of our analysis) but the general conditions where our model applies may be broad given that most traits examined in natural populations show genetic variation, including traits that mediate the outcome of social interactions among conspecifics (e.g., Hunt and Simmons 2002; Wilson et al. 2005). When social interactions are synergistic or epistatic in nature (as expected under our model), it is possible that the interactions themselves generate some frequency‐dependent selection that actively maintains variation (Queller 1985, 2011), which appears to be a common feature of loci with greenbeard properties (e.g., (Smukalla et al. 2008; Biernaskie et al. 2013; Heller et al. 2016; Gruenheit et al. 2017). Furthermore, the process of social coadaptation reduces the fitness cost of standing genetic variation (i.e., reduces the genetic load) because it coordinates variation to result in higher frequencies of interactions between genotypes that result in high fitness (and hence lower frequencies of those that lead to low fitness; (Wolf and Brodie III 1998; Wolf 2000b). This phenomenon is enhanced in the case where imprinting increases social coadaptation (eqs. 3a and 3b), and hence imprinting can potentially allow for even higher levels of segregating variation than would be expected for coadaptation of nonimprinted genes.

We have intentionally presented this coadaptation model in very general terms to emphasize its broad applicability. Consequently, this model could potentially apply to any trait(s) that mediate social interactions between conspecifics with consequences for fitness, regardless of the mechanism or the type of organism. For example, behavioral interactions between parents and offspring have well‐documented consequences for fitness, particularly in the context of resource provision and demand in organisms with high levels of parental care such as many mammals (e.g., mice, Hager and Johnstone 2003), birds (e.g., tree swallows, Hussell 1988), and insects (e.g., burrowing bugs, Agrawal et al. 2001; burying beetles, Lock et al. 2004). However, it could equally be relevant to other social interactions among conspecifics that are mediated by behavior (e.g., aggression traits in deer mice, Wilson et al. 2009; social dominance in red deer, Wilson et al. 2011), or to interactions mediated by biochemical mechanisms, such as those arising during pregnancy (see Haig 1996). The Coadaptation Theory may therefore be able to explain imprinting in a greater range of organisms and traits than is predicted by existing theories.

To illustrate the ways in which predictions from our coadaptation model differ from those of other theories of the evolution of imprinting, consider the scenario of communal care (allocare) in mammals, where selection from kin interactions appears a likely explanation for the occurrence of genomic imprinting (Ubeda and Gardner 2015; Wolf et al. 2015), especially for imprinted expression in the mammary (Cowley et al. 2014; Ubeda and Gardner 2015; Wolf et al. 2015). In rodents, where allocare is particularly well‐studied, communally nesting females are typically related, and the relatedness of the pups to their nurses is known to influence their fitness (König 1994). The coadaptation process can drive imprinting evolution in the context of allocare if the fitness of offspring with a given phenotype (and hence genotype) depends on the phenotype (and hence genotype) of their nurse(s), and offspring and their nurses are asymmetrically related through their matrigenic and patrigenic gene copies. Under these conditions, the Coadaptation Theory predicts that the individuals (offspring and/or nurses) will express the gene copy through which relatedness is higher (Table 2; Supporting Information). By contrast, the Kinship Theory predicts expression of the gene copy for which there is an inclusive fitness benefit from higher total expression of that particular gene. Therefore, data on relatedness asymmetries and fitness effects associated with allocare should provide critical tests to distinguish between different hypotheses. As an example, consider the relatedness patterns in communal nests that would be expected to drive the observed patterns of imprinting in the *Grb10* gene in the mammary gland of mice under the Coadaptation Theory versus the Kinship Theory (see also Cowley et al. 2014; Ubeda and Gardner 2015; Wolf et al. 2015). The Coadaptation Theory would predict that nurses are more related to alien offspring through their matrigenic than their patrigenic allele while the Kinship Theory would predict that nurses are more related to recipients of allocare through their patrigenic alleles (Úbeda and Gardner 2010, 2011, 2012), but both predictions depend critically on untested assumptions about properties of the gene and the communal care setting. Further details about the example of the *Grb10* gene and the logic behind these predictions are outlined in the Supporting Information.

**Table 2:** Predicted patterns of imprinting for coadapted traits expressed in offspring and nurses for different relatedness structures in communal nests

	Offspring‐Nurse Relatedness (***r***_*XY*_)				Pattern of Imprinting Favored	
Allocare Scenario	$r_{\mathrm{MM}}$	$r_{\mathrm{MP}}$	$r_{\mathrm{PM}}$	$r_{\mathrm{PP}}$	Offspring (*I*)	Nurses (*J*)
(i) Solitary nesting	½	½	0	0	Maternal	None
(ii) Offspring's mother and nurse are full‐siblings	¼	¼	0	0	Maternal	None
(iii) Offspring's father and nurse are full‐siblings	0	0	¼	¼	Paternal	None
(iv) Offspring's mother and nurse are maternal half‐siblings	¼	0	0	0	Maternal	Maternal
(v) Offspring's mother and nurse are paternal half‐siblings	0	¼	0	0	Maternal	Paternal
(vi) Offspring's father and nurse are maternal half‐siblings	0	0	¼	0	Paternal	Maternal
(vii) Offspring's father and nurse are paternal half‐siblings	0	0	0	¼	Paternal	Paternal
(viii) Offspring and nurse are maternal half‐siblings	½	0	0	0	Maternal	Maternal
(ix) Offspring and nurse are paternal half‐siblings	0	0	0	½	Paternal	Paternal

The nine scenarios differ in the pattern of relatedness of the offspring to the nurse through their two gene copies, which are captured in the four relatedness terms (given by the genetic identity coefficients, ***r***_***XY***_, where the first subscript refers to the gene copy being considered in the offspring and the second the gene copy in the nurse). The pattern of imprinting favored in the offspring corresponds to the pattern of expression of the locus (*I*) for the direct trait (*D_i_*) and the pattern favored in the nurse corresponds to the pattern of expression of the locus (*J*) for the social trait (*S*_*j*_). Possible patterns are: maternal = maternal expression, paternal = paternal expression, and none = ordinary biallelic expression (i.e., no selection for imprinting). Solitary nesting corresponds to the case of maternal care (i.e., nurse = mother) and is included for comparison. With variation in the patterns of relatedness in communal nests in nature, the pattern of expression expected to occur will be determined by the weighted average pattern of relatedness.

Thus, our model demonstrates that selection can favor imprinting because it enhances social coadaptation. However, whether this Coadaptation Theory or the Kinship Theory, or some other theory, explains the occurrence of imprinting at any genes remains unresolved because appropriate data needed to differentiate among theories are mostly unavailable at present (Ubeda and Gardner 2015; Wolf et al. 2015). Achieving such resolution is important because an understanding of the evolutionary pressures shaping imprinting across genes and species will provide critical insights into gene function and dysfunction. The Coadaptation Theory should play an important role in this process by providing a simple and clear framework for developing predictions and associated tests to distinguish between different theories for the evolution of imprinting. Implementation of these tests should be forthcoming as advances in genomic and transcriptomic technologies allow for high‐resolution characterization of imprinted genes and relatedness structures in a diverse array of taxa (Wang and Clark 2014).

Editor in Chief: J. Slate

Associate Editor: A. Gardner

## Supporting information


**Table S1**. Frequency of each allelic combination in interacting individuals in a randomly mating population.
**Table S2**. Frequencies of social interactions between each of the genotype combinations at the A locus in a randomly mating population.
**Table S3**. Frequency of each allelic combination at loci that modify imprinting of the A locus effect on (a) the direct trait (the “B” locus) and (b) the social trait (the “C” locus) in interacting individuals in a randomly mating population.
**Table S4**. Frequencies of social interactions between each of the genotype combinations at loci that modify imprinting of the effect of the A locus on (a) the direct trait (the “B” locus) and (b) the social trait (the “C” locus) in a randomly mating population.
