# Supplementary material for: The coadaptation theory for genomic imprinting
Source: Evol Lett. 2017 May 3;1(1):49–59. doi: 10.1002/evl3.5 (PMC6121825; doi:10.1002/evl3.5)
Supplement: Supplementary file 1 — Table S1. Frequency of each allelic combination in interacting individuals in a randomly mating population. Table S2. Frequencies of social interactions between each of the genotype combinations at the A locus in a randomly mating population. Table S3. Frequency of each allelic combination at loci that modify imprinting of the A locus effect on (a) the direct trait (the “B” locus) and (b) the social trait (the “C” locus) in interacting individuals in a randomly mating population. Table S4. Frequencies of social interactions between each of the genotype combinations at loci that modify imprinting of the effect of the A locus on (a) the direct trait (the “B” locus) and (b) the social trait (the “C” locus) in a randomly mating population. [file EVL3-1-49-s001.docx]

**Supplementary Material contents:**

**Supplementary Methods:** Evolution of imprinting through selection on imprinting modifier loci

**Illustrating evolution of imprinting through social coadaptation in communal nests**

**Tables S1 – S4**

**Supplementary Material:**

**Supplementary Methods: Evolution of imprinting through selection on imprinting modifier loci**

The basic assumptions and structure of the imprinting modifier models are provided in the main text. Here we provide further details of the model construction and derivation. For reference a list of model terms are provided in Table 1.

*Imprinting for the direct trait*

To understand the conditions that favour imprinting at the A locus for its effect on the direct trait we derive selection on a modifier allele that causes some pattern of imprinting. The phenotypic value of the direct trait (*D_i_*) associated with the ordered genotypes at the A locus for individuals that are homozygotes for the *B*_2_ allele at the B locus, which does not cause imprinting, match those for the case of *I* = 0 presented in the main text (see below). The *B*_1_ allele modifies these expected phenotypes associated with A locus genotypes by causing some pattern of imprinting, with the phenotypic value of the direct trait of a given A locus genotype being biased towards the gene copy that is not imprinted. The effect of the B locus on imprinting of the A locus effect on the direct trait is additive, with the *B*_1_ allele causing a pattern of imprinting given by *δ* (which is analogous to the parameter *I* defined in the main text). For example, following the logic outlined in the main text for the pattern of trait expression as a function of the imprinting parameter *I*, the phenotypic value of the direct trait for genotype *A*_1_*A*_2_*B*_1_*B*_1_ is ½[(1 + *δ*)*a_d_* – (1 – *δ*)*a_d_*] = *δa_d_*, while the phenotypic value for *A*_2_*A*_1_*B*_1_*B*_2_ is ½[– (1 + ½*δ*)*a_d_* + (1 – ½*δ*)*a_d_*] = –½*δa_d_*. Following the same logic, we define the matrix of the direct trait phenotypic values associated with the sixteen possible A-B locus genotype combinations by considering how the vector of direct trait phenotypic values (**D_A_**) changes for each of the four B locus genotypes to create a matrix of phenotypes for the sixteen two-locus genotypes, **D_AB_**, (where rows correspond to the four A locus genotypes, ordered as above, and columns to the four B locus genotypes, also ordered as above). The phenotypic values for the direct trait vary across the columns of the **D_AB_** matrix because the B locus genotype leads to different patterns of expression at the A locus across columns. We denote these individual column vectors **D_AB_**_(_*_kl_*_)_ (where the *k* and *l* subscripts indicate identity of the two B locus alleles). For individuals with the *B*_1_*B*_1_ genotype at the B locus (which imprints the A locus), **D_AB(_**_11)_ =[*a_d_*, *δa_d_*, ‒*δa_d_*, and ‒*a_d_*]^T^, while those with either the *B*_1_*B*_2_ or *B*_2_*B*_1_ genotypes (which cause intermediate imprinting at the A locus) are **D_AB(_**_12)_ = **D_AB(_**_21)_ = [*a_d_*, ½*δa_d_*, ‒½*δa_d_*, and ‒*a_d_*]^T^ and with the *B*_2_*B*_2_ (which do not imprint the A locus) are **D_AB(_**_22)_ = [*a_d_*, 0, 0, and ‒*a_d_*]^T^. These four column vectors are combined into the A-B two-locus matrix of direct trait phenotypic values: **D_AB_** = [**D_AB(_**_11)_, **D_AB(_**_12)_, **D_AB(_**_21)_, **D_AB(_**_22)_].

The phenotypic value of the social trait associated with A locus genotypes (**S_A_**) are unaffected by the B locus genotype, but we need to specify the matrix of social trait phenotypic values for the sixteen A-B two-locus genotypes to define fitness. The structure of the matrix of phenotypic values of the social trait associated with the A-B genotypes follows those defined above for direct trait, with the column vectors for the phenotypes associated with the four B locus genotypes being: **S_AB(_**_11)_ = **S_AB(_**_12)_ = **S_AB(_**_21)_ = **S_AB(_**_22)_ = [*a_s_*, *Ja_s_*, ‒*Ja_s_*, ‒*a_s_*]^T^. These four column vectors are combined into the two-locus matrix of social trait phenotypic values: **S_AB_** = [**S_AB_**_11_, **S_AB_**_12_, **S_AB_**_21_, **S_AB_**_22_]. Individual fitness for the two-locus genotypes of focal individuals as a function of the two-locus genotype of their partner (given by the vector **w_AB_**) follows the model in equation (1) in the main text, but for the two-locus system is defined as:

$\mathbf{w}_{\mathbf{AB}}\mathbf{=}1 + \psi\mathrm{vec}\left( \mathbf{S}_{\mathbf{AB}}\boldsymbol{\bigotimes D}_{\mathbf{AB}} \right)$. [M1]

Because the B locus is not linked to the A locus, the frequencies of the focal-partner genotype combinations for the sixteen two-locus genotype combinations (i.e., combinations of diploid A locus and B locus allelic combinations), **F_AB_**, are simply the products of the independent frequencies for the two loci. Therefore: $\mathbf{F}_{\mathbf{AB}}\mathbf{=}\mathrm{vec}\left( \mathbf{F}_{\mathbf{B}}\mathbf{F}_{\mathbf{A}}^{T} \right)$, where **F_A_** and **F_B_** are the vectors of frequencies of genotype combinations for the A locus and the B locus (defined in Tables S2 and S4a respectively).

To derive the expression for evolutionary change in the frequency of the modifier allele, Δ*x*_1_, we first need to define the vector, **N_AB_**, that contains the proportion of *B*_1_ alleles in the focal individual’s genotype for all 256 possible focal-partner two-locus genotype combinations (e.g., a focal individual with genotype *B*_1_*B*_1_ at the B locus would have a value of 1, regardless of their A locus genotype or the two-locus genotype of their partner), with entries ordered as in the vectors **w_AB_** and **F_AB_**. Because of the simplicity and size of this matrix we do not write out all entries here. The evolutionary change in the frequency of the *B*_1_ allele is defined by the covariance between the proportion of an individual’s alleles at the B locus that are *B*_1_ with its relative fitness ($w_{ij}/\bar{w}$), which gives the change in the frequency of the *B*_1_ allele, ([Price, 1970](#_ENREF_15)): $\mathrm{cov}\left( B_{1},w_{ij} \right)\bar{w}=\Delta x_{1}\bar{w}=\mathbf{F}_{\mathbf{AB}}\boldsymbol{\cdot}\left[ \left( \mathbf{w}_{\mathbf{AB}}\boldsymbol{-}\bar{\boldsymbol{w}} \right)\left( \mathbf{N}_{\mathbf{AB}}\boldsymbol{-}\boldsymbol{x}_{\boldsymbol{1}} \right) \right]$, which equals:

$\Delta x_{1}\bar{w}=\frac{1}{4}{(x}_{1}x_{2})a_{d}a_{s}p_{1}p_{2}\psi\left[ \left( 1+J \right)\left( r_{MM}-r_{PM} \right)+\left( 1-J \right)\left( r_{MP}-r_{PP} \right) \right]$ [M2]

The RHS of this equation has three components: 1) the effect of the modifier allele on imprinting, *δ*, 2) the degree of allelic variation at the B locus ($x_{1}x_{2}$) and 3) the pattern of selection favouring imprinting, which is $a_{d}a_{s}p_{1}p_{2}\psi\left[ \left( 1+J \right)\left( r_{MM}-r_{PM} \right)+\left( 1-J \right)\left( r_{MP}-r_{PP} \right) \right]$. This last component defines the selection gradient on imprinting, *β_I_*, and therefore is critically important because it provides a general statement of the pattern of selection favouring imprinting (see eqn. 5). Whether selection for imprinting favours a particular modifier allele simply depends on the direction of imprinting it causes (i.e., whether the sign of *δ* matches the sign of *β_I_*, which results in an increase in the frequency of the modifier allele, or vice versa). Consequently, the evolutionary change in the frequency of the imprinting modifier can be expressed simply as $\Delta x_{1}\bar{w}=\frac{1}{4}{(x}_{1}x_{2})\beta_{I}$.

*Imprinting for the social trait*

To understand the conditions that favour imprinting at the A locus for its effect on the social trait we follow the approach outlined above for the direct trait. However, the structure of the model for imprinting of the A locus effect on the social trait has a critical difference from imprinting for the effect on the direct trait; while the C locus genotype of an individual causes imprinting of the effect of the A locus on their expression of the social trait, it is the C locus genotype of an individual’s social partner that can affect their fitness (by altering the social environment they experience). Therefore, the influence of imprinting caused by a modifier allele at the C locus on the association between the A locus genotype and the social trait does not mediate the relationship between an individuals genotype and their own fitness, but rather, the relationship between their genotype and the fitness of their social partner. To capture this relationship we first model the influence of the C locus genotype on the association between the genotype of social partners and their value for the social trait and then translate this social influence on fitness into selection on the modifier allele.

The phenotypic value of the social trait (*S_j_*) associated with the ordered genotypes at the A locus for individuals that are homozygotes for the *C*_2_ allele at the C locus, which does not cause imprinting, match those for the case of *J* = 0 presented in the main text (see below). The *C*_1_ allele modifies these expected phenotypes associated with A locus genotypes by causing some pattern of imprinting, with the phenotypic value of the social trait for a given A locus genotype being biased towards the gene copy that is not imprinted. The effect of the C locus on imprinting of the A locus for its effect on the social trait is additive, with the *C*_1_ allele causing a pattern of imprinting given by *σ* (which is analogous to the parameter *J* defined in the main text). Following the example from the B locus case, the phenotypic value of the social trait for genotype *A*_1_*A*_2_*C*_1_*C*_1_ is ½[(1 + *σ*)*a_s_* – (1 – *σ*)*a_s_*] = *σa_s_*, while the phenotype for *A*_2_*A*_1_*C*_1_*C*_2_ is ½[– (1 + ½*σ*)*a_d_* + (1 – ½*σ*)*a_s_*] = –½*δσ_s_*. Following the same logic, we define the matrix of social trait phenotypic values associated with the sixteen possible A-C locus genotype combinations by considering how the vector of phenotypic values for the social trait (**S_A_**) changes for each of the four C locus genotypes to create a matrix of phenotypic values for the sixteen two-locus genotypes, **S_AC_**, (where rows correspond to the four A locus genotypes, ordered as above, and columns to the four C locus genotypes, also ordered as above). The phenotypic values for the social trait vary across the columns of the **S_AC_** matrix because the C locus genotype leads to different patterns of expression at the A locus across columns. We denote these individual column vectors **S_AC_**_(_*_kl_*_)_, (where the *k* and *l* subscripts indicates the pair of C locus alleles). For individuals with the *C*_1_*C*_1_ genotype at the C locus (which imprints the A locus effect on the social trait), **S_AC(_**_11)_ =[*a_s_*, *σa_s_*, ‒*σa_s_*, and ‒*a_s_*]^T^, while those with either the *C*_1_*C*_2_ or *C*_2_*C*_1_ genotypes (which cause intermediate imprinting of the effect of the A locus on the social trait) are **S_AC(_**_12)_ = **S_AC(_**_21)_ = [*a_s_*, ½*σa_s_*, ‒½*σa_s_*, and ‒*a_s_*]^T^ and with the *C*_2_*C*_2_ (which do not cause imprinting at the A locus) are **S_AC(_**_22)_ = [*a_s_*, 0, 0, and ‒*a_s_*]^T^. These four column vectors are combined into the two-locus matrix of social trait phenotypic values: **S_AC_** = [**S_AC(_**_11)_, **S_AC(_**_12)_, **S_AC(_**_21)_, **S_AC(_**_22)_].

The phenotypic value of the direct trait associated with A locus genotypes (**D_A_**) are unaffected by an individual’s C locus genotype, but we need to specify the matrix of direct trait phenotypic values for the sixteen A-C two-locus genotypes to define individual fitness. The structure of the matrix of direct trait phenotypic values follows that defined above for the social trait, with the column vectors for the direct trait phenotypic values associated with the four C locus genotypes being: **D_AC(_**_11)_ = **D_AC(_**_12)_ = **D_AC(_**_21)_ = **D_AC(_**_22)_ = [*a_d_*, *Ia_d_*, ‒*Ia_d_*, ‒*a_d_*]^T^. These four column vectors are combined into the A-C two-locus matrix of direct trait phenotypic values: **D_AC_** = [**D_AC_**_11_, **D_AC_**_12_, **D_AC_**_21_, **D_AC_**_22_]. Individual fitness for the two-locus genotypes of focal individuals as a function of the two-locus genotype of their partner (given by the vector **w_AC_**) follows the model in equation (M1), but for the A-C two-locus system are defined as: $\mathbf{w}_{\mathbf{AC}}\mathbf{=}1 + \psi\mathrm{vec}\left( \mathbf{S}_{\mathbf{AC}}\boldsymbol{\bigotimes D}_{\mathbf{AC}} \right)$.

Because the C locus is not linked to the A locus, the frequencies of the focal-partner genotype combinations for the sixteen two-locus genotype combinations (i.e., combinations of diploid A locus and C locus allelic combinations), **F_AC_**, are simply the products of the independent frequencies for the two loci. Therefore: $\mathbf{F}_{\mathbf{AC}}\mathbf{=}\mathrm{vec}\left( \mathbf{F}_{\mathbf{C}}\mathbf{F}_{\mathbf{A}}^{T} \right)$, where **F_A_** and **F_C_** are the vectors of frequencies of genotype combinations for the A locus and the C locus (defined in Tables S2 and S4b respectively).

To examine evolution of imprinting of the A locus for its effect on the social trait, we derive the covariance between the proportion of an individual’s alleles at the C locus that are *C*_1_ with its relative fitness ($w_{ij}/\bar{w}$),which gives the change in the frequency of the *C*_1_ allele, Δ*y*_1_ ([Price, 1970](#_ENREF_15)). To do so, we define a vector, **N_AC_**, that contains the proportion of *C*_1_ alleles in the focal individual’s genotype for all 256 possible focal-partner two-locus genotype combinations (following the B locus derivation), with entries ordered as in the vectors **w_AC_** and **F_AC_**. The evolutionary change in the frequency of the *C*_1_ allele is therefore defined by: $\mathrm{cov}\left( C_{1},w_{ij} \right)\bar{w}=\Delta y_{1}\bar{w}=\mathbf{F}_{\mathbf{AC}}\boldsymbol{\cdot}\left[ \left( \mathbf{w}_{\mathbf{AC}}\boldsymbol{-}\bar{\boldsymbol{w}} \right)\left( \mathbf{N}_{\mathbf{AC}}\boldsymbol{-}\boldsymbol{y}_{\boldsymbol{1}} \right) \right]$, which equals:

$\Delta y_{1}\bar{w}=\frac{1}{4}\sigma(y_{1}y_{2})\phi_{GG^{'}}a_{d}a_{s}p_{1}p_{2}\psi[\left( 1+I \right)\left( r_{MM}-r_{MP} \right)+\left( 1-I \right)\left( r_{PM}-r_{PP} \right)]$ [M3]

As with the evolution of the B locus, the RHS of this equation is comprised of three components: 1) the effect of the modifier allele on imprinting, *σ*, 2) the degree of allelic variation at the C locus ($y_{1}y_{2}$) and 3) the pattern of selection favouring imprinting, which is $\phi_{GG^{'}}a_{d}a_{s}p_{1}p_{2}\psi[\left( 1+I \right)\left( r_{MM}-r_{MP} \right)+\left( 1-I \right)\left( r_{PM}-r_{PP} \right)]$. As with imprinting for the direct trait, this selection component defines the selection gradient, *β_J_* (eqn. 6), which gives the conditions under which selection favours imprinting for the effect of the A locus on the social trait. Whether selection for imprinting favours a particular modifier allele simply depends on the direction of imprinting it causes (i.e., whether the sign of *σ* matches the sign of *β_J_*). Consequently, the evolutionary change in the frequency of the imprinting modifier can be expressed as $\Delta y_{1}\bar{w}=\frac{1}{4}\sigma{(y}_{1}y)\beta_{J}$. Note that selection on a modifier of imprinting of the effect of the A locus on the social trait differs from that for the direct trait (eqn. M2) in that it is weighted by the genetic coefficient of kinship ($\phi_{GG^{'}}$).

**Illustrating evolution of imprinting through social coadaptation in communal nests**

To understand the predictions and utility of our Coadaptation Theory, we illustrate the model structure and predictions in the context of interactions between offspring and nurses in species that engage in communal care (‘allocare’). Allocare is widespread in mammals, occurring across most major taxonomic groups ([MacLeod and Lukas, 2014](#_ENREF_12), [Packer et al., 1992](#_ENREF_14)), and typically includes ‘allonursing’ in which females provide milk to offspring that are not their own (‘alien’ offspring) when living in communal nests with other females ([Hayes, 2000](#_ENREF_7), [Packer et al., 1992](#_ENREF_14), [MacLeod and Lukas, 2014](#_ENREF_12)). Allonursing can have important consequences for offspring fitness ([König, 1994a](#_ENREF_10), [König, 1994b](#_ENREF_11), [Hayes and Solomon, 2004](#_ENREF_8)), and, as expected for coadaptation, offspring fitness has been found to be higher when communally nursing females are close relatives ([König, 1994a](#_ENREF_10), [König, 1994b](#_ENREF_11)). We focus on the structure of allonursing in mice, where communal nesting is common and particularly well characterized ([Hayes, 2000](#_ENREF_7), [König, 1994a](#_ENREF_10), [König, 1994b](#_ENREF_11)).

Communal nests are formed by two or more nursing females. The frequency with which each offspring-nurse A locus genotype combination occurs is denoted $f_{ij}$ (where *i* indicates the offspring genotype and *j* the nurse genotype), and depends upon the relatedness of offspring and nurses within the nest (Table S2). We assume that, once a communal nest is formed, females sharing a nest nurse offspring indiscriminately (such that the frequencies with which females of different genotypes nest together determines the probabilities that offspring experience each nurse genotype), which is consistent with empirical data from mice ([König, 1989](#_ENREF_9), [Manning et al., 1995](#_ENREF_13)).

We can assume that the A locus affects traits expressed in offspring (the direct trait) and in mothers (in their role as nurses, which represents the social trait) ([cf. Wolf and Hager, 2006](#_ENREF_26)) in the context of communal care. Such pleiotropic expression of imprinted genes early in development and in adults has been observed for several imprinted genes (see [Cowley et al., 2014](#_ENREF_4), [Garfield et al., 2011](#_ENREF_6), [Stringer et al., 2014](#_ENREF_17), [Stringer et al., 2012](#_ENREF_18), [Renfree et al., 2013](#_ENREF_16)), and is consistent with the observation that imprinting is often tissue and developmental-stage specific ([Tycko and Morison, 2002](#_ENREF_19)).

To develop an intuitive understanding of the process and predictions of this model we explore a set of biologically realistic scenarios of relatedness that may be observed in communal nests. These scenarios are likely to result from specific patterns of migration and population viscosity (especially sex-specific patterns):

1. Females nest individually and only nurse their own offspring. This scenario matches that assumed in the special case of the Maternal-Offspring Coadaptation Theory ([Wolf and Hager, 2006](#_ENREF_26)).
2. The offspring’s mother and its nurse are full-siblings. This corresponds to a situation where female offspring from a litter remain in the local area and rear their offspring communally.
3. The offspring’s father and the nurse are full siblings. This corresponds to a situation where offspring of both sexes from a litter remain in the local area and mate.
4. The offspring’s mother and the nurse are maternal half-siblings. This could arise if females mate with multiple males, such that offspring within or among litters of the same mother have different fathers. Female offspring from the same mother then remain in the local area and rear their offspring communally (as in scenario ii).
5. The offspring’s mother and the nurse are paternal half-siblings. This could arise if males mate with multiple females (perhaps due to male territoriality), and then the female offspring from multiple litters (sired by the same father) remain in the local area and rear their offspring communally (as in scenarios ii and iv)
6. The offspring’s father and the nurse are maternal half-siblings. This could arise if females mate with multiple males, such that offspring within or among litters of the same mother have different fathers (as in scenario iv), and then offspring of both sexes from a litter remain locally to mate and produce offspring (after which males may disperse), as in (iii).
7. The offspring’s father and the nurse are paternal half-siblings. This could arise if males mate with groups of females in a nest (as in scenario v), and then offspring of both sexes from the same father remain locally and produce offspring (after which males may disperse), as in (iii) and (vi).
8. The offspring and the nurse are maternal half-siblings. This could arise if a female has multiple litters sired by different males, and daughters from previous litters remain locally and rear and nurse their own offspring alongside those of their mother.
9. The offspring and the nurse are paternal half-siblings. This could arise if males sire litters by multiple females in a nest, and daughters from previous litters remain locally and rear and nurse their own offspring alongside other females whose offspring are sired by their father.

The patterns of imprinted expression expected to evolve in offspring (i.e., for the direct trait) and nurses (i.e., for the social trait) are defined by the selection gradients given in equations (5) and (6), and are summarized for each scenario in Table 1. As demonstrated by the Maternal-Offspring Coadaptation Theory, we find that interactions between mothers and their offspring (scenario i) can favour the evolution of maternal expression in offspring ([Wolf and Hager, 2006](#_ENREF_26)) (Table 1). Because maternal-offspring interactions are expected to occur in a communal nest under all scenarios, we ignore this element when evaluating other scenarios, but note that the pattern of selection observed under scenarios (ii) – (ix) is *in addition* to that observed due to selection for mother-offspring coadaptation. Furthermore, we emphasize that this list of scenarios is not exhaustive, nor are the scenarios mutually exclusive. Where multiple relationships between offspring and nurses exist within communal nests, the pattern of expression favoured will be determined by the weighted average pattern of relatedness.

In both offspring and nurses, imprinted expression is favoured whenever the gene copies in one partner are asymmetrically related to a given allele in the other partner (Table 1). In scenarios (i) – (iii), selection does not favour imprinted expression in the nurse because both gene copies in the nurse are equally related to the offspring. Imprinted expression in the offspring is favoured under these scenarios, with expression of the gene copy through which they are more related to the nurse. Under the remaining scenarios considered here, coadaptation favours imprinted expression in both offspring and nurses (always with expression of the gene copy through which they are more related to the other partner), as long as the gene copy in the partner with which the gene copies in the focal individual are asymmetrically related is not silenced. We note that under an additional scenario, where offspring and nurses are full-siblings, imprinted expression is not favoured in either partner (because there is no overall relatedness asymmetry), *unless* there is already imprinted expression in one of the partners, which could arise through coadaptation with a different type of relative e.g. maternal-offspring coadaptation ([Wolf and Hager, 2006](#_ENREF_26)). Selection will then favour a matching pattern of imprinted expression in the other partner. This is similar to the process that is predicted to drive the evolution of matching patterns of imprinted expression among interacting genes within a genome ([Wolf, 2013](#_ENREF_24)).

In all cases, the pattern of imprinting is favoured because it increases coadaptation, which is captured in the covariance between offspring and nurses (eqn. 3). This prediction is consistent with the empirical observation that survival of pups to weaning is significantly higher when communally nursing females are close relatives ([König, 1994a](#_ENREF_10), [König, 1994b](#_ENREF_11)). Where imprinted expression is favoured by selection, the strength of selection favouring imprinting increases with the relatedness of offspring and nurses, the degree to which the relevant gene copy is expressed in the other partner, the amount of genetic variation at the A locus, and the effect of the social interaction on fitness (𝜓).

In this example, we have shown that the structure of relatedness within the group (i.e. the matrigenic vs patrigenic relationship between offspring and allonursing females) determines the patterns of imprinting that are expected to evolve by coadaptation (Table 1). Our model generates clear predictions about the relatedness structures that are expected given observed expression patterns at imprinted loci, and vice versa, which could be tested in natural populations. Studies of several communally nesting species have shown that coefficients of relatedness among mothers within nests (and therefore between offspring and nurses) are typically significantly greater, on average, than among random members of the population ([Auclair et al., 2014](#_ENREF_1), [Ebensperger et al., 2004](#_ENREF_5)), but measurements of relatedness generally do not differentiate relatedness through the matrigenic and patrigenic alleles (i.e., they average across these two relatedness components). Consequently, it is difficult to assess whether natural populations show the relatedness asymmetries that fit the Kinship or Coadaptation Theories. Relatedness of individuals within nests is determined by factors such as sex-specific reproduction and dispersal behaviours, and is therefore likely to show considerable variation across the wide array of mammal species that engage in communal nesting ([MacLeod and Lukas, 2014](#_ENREF_12), [Packer et al., 1992](#_ENREF_14)). Comparative studies of imprinting patterns at genes across species with different relatedness structures in communal nests would provide data that could be used to evaluate support for the different theories of imprinting.

The mouse gene *Grb10* provides a compelling case study because of its widespread pleiotropic effects and peculiar pattern of imprinting ([Garfield et al., 2011](#_ENREF_6), [Cowley et al., 2014](#_ENREF_4)), which has led to an unresolved debate over the evolutionary processes shaping imprinting of the gene ([Wolf et al., 2015](#_ENREF_25), [Ubeda and Gardner, 2015](#_ENREF_20)). *Grb10* is maternally expressed in the placenta and developing embryo, where it acts to reduce growth ([Charalambous et al., 2010](#_ENREF_2), [Charalambous et al., 2003](#_ENREF_3)), and in nursing pups, where it reduces nutrient demand ([Cowley et al., 2014](#_ENREF_4)). It is also maternally expressed in the mammary gland of mothers, where it increases nutrient supply during lactation ([Cowley et al., 2014](#_ENREF_4)). The patterns of imprinting and effect in the embryo and pup are consistent with the predictions of the Kinship Theory, while the patterns in the mammary are not direct predictions of the Kinship Theory applied to parents and offspring, but could be consistent with the Kinship Theory when extended beyond parent-offspring interactions, depending on the pattern of relatedness in communally nesting mice in nature ([Ubeda and Gardner, 2015](#_ENREF_20)). Matching expression of *Grb10* in both mother and pup is required for ‘normal’ body size and body composition (lean/fat ratio) of pups, implying complementary roles for *Grb10* in mothers and offspring ([Cowley et al., 2014](#_ENREF_4)), which is consistent with the expectations of the Maternal-Offspring Coadaptation Theory ([Wolf and Hager, 2006](#_ENREF_26)), while the pattern of expression in the mammary of nursing females may be consistent with our general Coadaptation Theory depending on patterns of relatedness in communal nests. Most importantly, the Kinship and Coadaptation Theories would predict different patterns of relatedness in communal nests given the pattern of imprinting in the mammary, offering an opportunity to differentiate the models with data ([Wolf et al., 2015](#_ENREF_25)). The Coadaptation Theory predicts that nurses are more related to alien offspring through their matrigenic than their patrigenic allele (e.g. if nursing females in a nest were maternal half-sibs, or mothers and daughters), and that variation at the gene has complementary effects in pups and nurses. By contrast, the Kinship Theory ([Úbeda and Gardner, 2011a](#_ENREF_22), [Úbeda and Gardner, 2011b](#_ENREF_23), [Úbeda and Gardner, 2010](#_ENREF_21)) predicts that nurses are more related to recipients of allocare through their patrigenic alleles (e.g. if nursing females in a nest were paternal half-sibs), and that the level of gene expression modulates a tradeoff between provision of maternal care (females nursing their own offspring) and allonursing provided to the offspring of their nest-mates ([Ubeda and Gardner, 2015](#_ENREF_20)). Determining the nature of relatedness and resource-provisioning in communal nests would therefore allow support for these contrasting theories of the evolution of imprinting to be evaluated.

**Table S1. Frequency of each allelic combination in interacting individuals in a randomly mating population.** The combinations are named according the parent of origin of the alleles (where ‘*M*’ indicates the matrigenic allele and ‘*P*’ the patrigenic allele), with the first position corresponding to the allele in the focal individual and the second position the allele in the social partner. The subscripts indicate the identify of the allele (where *A*1 = *A_1_* and *A*2 = *A_2_*). For example, the combination *M*_A1_*P*_A2_ indicates that the focal individual’s matrigenic allele is *A_1_* and their social partner’s patrigenic allele is *A_2_*. Frequencies of each combination are determined by the values of the appropriate relatedness term and the allele frequencies at the locus.

| **Allelic combination in focal individuals and social partners** | **Frequency** |
| --- | --- |
| *M*_A1_*M*_A1_ | $p_{1}\left( r_{MM}+\left( 1-r_{MM} \right)p_{1} \right)$ |
| *M*_A1_*M*_A2_ | $p_{1}\left( 1-r_{MM} \right)p_{2}$ |
| *M*_A2_*M*_A1_ | $p_{2}\left( 1-r_{MM} \right)p_{1}$ |
| *M*_A2_*M*_A2_ | $p_{2}\left( r_{MM}+\left( 1-r_{MM} \right)p_{2} \right)$ |
|  |  |
| *M*_A1_*P*_A1_ | $p_{1}\left( r_{MP}+\left( 1-r_{MP} \right)p_{1} \right)$ |
| *M*_A1_*P*_A2_ | $p_{1}\left( 1-r_{MP} \right)p_{2}$ |
| *M*_A2_*P*_A1_ | $p_{2}\left( 1-r_{MP} \right)p_{1}$ |
| *M*_A2_*P*_A2_ | $p_{2}\left( r_{MP}+\left( 1-r_{MP} \right)p_{2} \right)$ |
|  |  |
| *P*_A1_*M*_A1_ | $p_{1}\left( r_{PM}+\left( 1-r_{PM} \right)p_{1} \right)$ |
| *P*_A1_*M*_A2_ | $p_{1}\left( 1-r_{PM} \right)p_{2}$ |
| *P*_A2_*M*_A1_ | $p_{2}\left( 1-r_{PM} \right)p_{1}$ |
| *P*_A2_*M*_A2_ | $p_{2}\left( r_{PM}+\left( 1-r_{PM} \right)p_{2} \right)$ |
|  |  |
| *P*_A1_*P*_A1_ | $p_{1}\left( r_{PP}+\left( 1-r_{PP} \right)p_{1} \right)$ |
| *P*_A1_*P*_A2_ | $p_{1}\left( 1-r_{PP} \right)p_{2}$ |
| *P*_A2_*P*_A1_ | $p_{2}\left( 1-r_{PP} \right)p_{1}$ |
| *P*_A2_*P*_A2_ | $p_{2}\left( r_{PP}+\left( 1-r_{PP} \right)p_{2} \right)$ |

**Table S2.** **Frequencies of social interactions between each of the genotype combinations at the A locus in a randomly mating population.** Frequencies of genotype interactions are a function of the frequencies of each allelic combination shown in Extended Data Table 1. Genotypes are listed with the matrigenic allele first, followed by the patrigenic allele. The frequency values in the last column define the interaction frequencies vector for the A locus, **F_A_**.

| **Focal individual genotype** | **Social partner genotype** | **Frequency**  **(F_A_)** |
| --- | --- | --- |
| *A*_1_*A*_1_ | *A*_1_*A*_1_ | ½ (*M*_A1_*M*_A1_ *P*_A1_*P*_A1_) + ½ (*M*_A1_*P*_A1_  *P*_A1_*M*_A1_) |
| *A*_1_*A*_1_ | *A*_1_*A*_2_ | ½ (*M*_A1_*M*_A1_ *P*_A1_*P*_A2_) + ½ (*M*_A1_*P*_A2_  *P*_A1_*M*_A1_) |
| *A*_1_*A*_1_ | *A*_2_*A*_1_ | ½ (*M*_A1_*M*_A2_ *P*_A1_*P*_A1_) + ½ (*M*_A1_*P*_A1_  *P*_A1_*M*_A2_) |
| *A*_1_*A*_1_ | *A*_2_*A*_2_ | ½ (*M*_A1_*M*_A2_ *P*_A1_*P*_A2_) + ½ (*M*_A1_*P*_A2_  *P*_A1_*M*_A2_) |
|  |  |  |
| *A*_1_*A*_2_ | *A*_1_*A*_1_ | ½ (*M*_A1_*M*_A1_ *P*_A2_*P*_A1_) + ½ (*M*_A1_*P*_A1_  *P*_A2_*M*_A1_) |
| *A*_1_*A*_2_ | *A*_1_*A*_2_ | ½ (*M*_A1_*M*_A1_ *P*_A2_*P*_A2_) + ½ (*M*_A1_*P*_A2_  *P*_A2_*M*_A1_) |
| *A*_1_*A*_2_ | *A*_2_*A*_1_ | ½ (*M*_A1_*M*_A2_ *P*_A2_*P*_A1_) + ½ (*M*_A1_*P*_A1_  *P*_A2_*M*_A2_) |
| *A*_1_*A*_2_ | *A*_2_*A*_2_ | ½ (*M*_A1_*M*_A2_ *P*_A2_*P*_A2_) + ½ (*M*_A1_*P*_A2_  *P*_A2_*M*_A2_) |
|  |  |  |
| *A*_2_*A*_1_ | *A*_1_*A*_1_ | ½ (*M*_A2_*M*_A1_ *P*_A1_*P*_A1_) + ½ (*M*_A2_*P*_A1_  *P*_A1_*M*_A1_) |
| *A*_2_*A*_1_ | *A*_1_*A*_2_ | ½ (*M*_A2_*M*_A1_ *P*_A1_*P*_A2_) + ½ (*M*_A2_*P*_A2_  *P*_A1_*M*_A1_) |
| *A*_2_*A*_1_ | *A*_2_*A*_1_ | ½ (*M*_A2_*M*_A2_ *P*_A1_*P*_A1_) + ½ (*M*_A2_*P*_A1_  *P*_A1_*M*_A2_) |
| *A*_2_*A*_1_ | *A*_2_*A*_2_ | ½ (*M*_A2_*M*_A2_ *P*_A1_*P*_A2_) + ½ (*M*_A2_*P*_A2_  *P*_A1_*M*_A2_) |
|  |  |  |
| *A*_2_*A*_2_ | *A*_1_*A*_1_ | ½ (*M*_A2_*M*_A1_ *P*_A2_*P*_A1_) + ½ (*M*_A2_*P*_A1_  *P*_A2_*M*_A1_) |
| *A*_2_*A*_2_ | *A*_1_*A*_2_ | ½ (*M*_A2_*M*_A1_ *P*_A2_*P*_A2_) + ½ (*M*_A2_*P*_A2_  *P*_A2_*M*_A1_) |
| *A*_2_*A*_2_ | *A*_2_*A*_1_ | ½ (*M*_A2_*M*_A2_ *P*_A2_*P*_A1_) + ½ (*M*_A2_*P*_A1_  *P*_A2_*M*_A2_) |
| *A*_2_*A*_2_ | *A*_2_*A*_2_ | ½ (*M*_A2_*M*_A2_ *P*_A2_*P*_A2_) + ½ (*M*_A2_*P*_A2_  *P*_A2_*M*_A2_) |

**Table S3. Frequency of each allelic combination at loci that modify imprinting of the A locus effect on (a) the direct trait (the ‘B’ locus) and (b) the social trait (the ‘C’ locus) in interacting individuals in a randomly mating population.** The combinations are named according the parent of origin of the alleles (where ‘*M*’ indicates the matrigenic allele and ‘*P*’ the patrigenic allele), with the first position corresponding to the allele in the focal individual and the second position the allele in the social partner (cf. Extended Data Table 1).

1. Frequency of allelic combinations at the B locus.

| **Allelic combination in focal individuals and social partners** | **Frequency** |
| --- | --- |
| *M*_B1_*M*_B1_ | $x_{1}\left( r_{MM}+\left( 1-r_{MM} \right)x_{1} \right)$ |
| *M*_B1_*M*_B2_ | $x_{1}\left( 1-r_{MM} \right)x_{2}$ |
| *M*_B2_*M*_B1_ | $x_{2}\left( 1-r_{MM} \right)x_{1}$ |
| *M*_B2_*M*_B2_ | $x_{2}\left( r_{MM}+\left( 1-r_{MM} \right)x_{2} \right)$ |
|  |  |
| *M*_B1_*P*_B1_ | $x_{1}\left( r_{MP}+\left( 1-r_{MP} \right)x_{1} \right)$ |
| *M*_B1_*P*_B2_ | $x_{1}\left( 1-r_{MP} \right)x_{2}$ |
| *M*_B2_*P*_B1_ | $x_{2}\left( 1-r_{MP} \right)x_{1}$ |
| *M*_B2_*P*_B2_ | $x_{2}\left( r_{MP}+\left( 1-r_{MP} \right)x_{2} \right)$ |
|  |  |
| *P*_B1_*M*_B1_ | $x_{1}\left( r_{PM}+\left( 1-r_{PM} \right)x_{1} \right)$ |
| *P*_B1_*M*_B2_ | $x_{1}\left( 1-r_{PM} \right)x_{2}$ |
| *P*_B2_*M*_B1_ | $x_{2}\left( 1-r_{PM} \right)x_{1}$ |
| *P*_B2_*M*_B2_ | $x_{2}\left( r_{PM}+\left( 1-r_{PM} \right)x_{2} \right)$ |
|  |  |
| *P*_B1_*P*_B1_ | $x_{1}\left( r_{PP}+\left( 1-r_{PP} \right)x_{1} \right)$ |
| *P*_B1_*P*_B2_ | $x_{1}\left( 1-r_{PP} \right)x_{2}$ |
| *P*_B2_*P*_B1_ | $x_{2}\left( 1-r_{PP} \right)x_{1}$ |
| *P*_B2_*P*_B2_ | $x_{2}\left( r_{PP}+\left( 1-r_{PP} \right)x_{2} \right)$ |

1. Frequency of allelic combinations at the C locus.

| **Allelic combination in focal individuals and social partners** | **Frequency** |
| --- | --- |
| *M*_C1_*M*_C1_ | $y_{1}\left( r_{MM}+\left( 1-r_{MM} \right)y_{1} \right)$ |
| *M*_C1_*M*_C2_ | $y_{1}\left( 1-r_{MM} \right)y_{2}$ |
| *M*_C2_*M*_C1_ | $y_{2}\left( 1-r_{MM} \right)y_{1}$ |
| *M*_C2_*M*_C2_ | $y_{2}\left( r_{MM}+\left( 1-r_{MM} \right)y_{2} \right)$ |
|  |  |
| *M*_C1_*P*_C1_ | $y_{1}\left( r_{MP}+\left( 1-r_{MP} \right)y_{1} \right)$ |
| *M*_C1_*P*_C2_ | $y_{1}\left( 1-r_{MP} \right)y_{2}$ |
| *M*_C2_*P*_C1_ | $y_{2}\left( 1-r_{MP} \right)y_{1}$ |
| *M*_C2_*P*_C2_ | $y_{2}\left( r_{MP}+\left( 1-r_{MP} \right)y_{2} \right)$ |
|  |  |
| *P*_C1_*M*_C1_ | $y_{1}\left( r_{PM}+\left( 1-r_{PM} \right)y_{1} \right)$ |
| *P*_C1_*M*_C2_ | $y_{1}\left( 1-r_{PM} \right)y_{2}$ |
| *P*_C2_*M*_C1_ | $y_{2}\left( 1-r_{PM} \right)y_{1}$ |
| *P*_C2_*M*_C2_ | $y_{2}\left( r_{PM}+\left( 1-r_{PM} \right)y_{2} \right)$ |
|  |  |
| *P*_C1_*P*_C1_ | $y_{1}\left( r_{PP}+\left( 1-r_{PP} \right)y_{1} \right)$ |
| *P*_C1_*P*_C2_ | $y_{1}\left( 1-r_{PP} \right)y_{2}$ |
| *P*_C2_*P*_C1_ | $y_{2}\left( 1-r_{PP} \right)y_{1}$ |
| *P*_C2_*P*_C2_ | $y_{2}\left( r_{PP}+\left( 1-r_{PP} \right)y_{2} \right)$ |

**Table S4.** **Frequencies of social interactions between each of the genotype combinations at loci that modify imprinting of the effect of the A locus on (a) the direct trait (the ‘B’ locus) and (b) the social trait (the ‘C’ locus) in a randomly mating population.** Frequencies of genotype interactions are a function of the frequencies of each allelic combination at each locus shown in Extended Data Tables 3 (a) and (b). Genotypes are listed with the matrigenic allele first, followed by the patrigenic allele.

1. Frequencies of social interactions between genotype combinations at the B locus. The frequency values in the last column define the interaction frequencies vector for the B locus, **F_B_**.

| **Focal individual genotype** | **Social partner genotype** | **Frequency**  **(F_B_)** |
| --- | --- | --- |
| *B*_1_*B*_1_ | *B*_1_*B*_1_ | ½ (*M*_B1_*M*_B1_ *P*_B1_*P*_B1_) + ½ (*M*_B1_*P*_B1_  *P*_B1_*M*_B1_) |
| *B*_1_*B*_1_ | *B*_1_*B*_2_ | ½ (*M*_B1_*M*_B1_ *P*_B1_*P*_B2_) + ½ (*M*_B1_*P*_B2_  *P*_B1_*M*_B1_) |
| *B*_1_*B*_1_ | *B*_2_*B*_1_ | ½ (*M*_B1_*M*_B2_ *P*_B1_*P*_B1_) + ½ (*M*_B1_*P*_B1_  *P*_B1_*M*_B2_) |
| *B*_1_*B*_1_ | *B*_2_*B*_2_ | ½ (*M*_B1_*M*_B2_ *P*_B1_*P*_B2_) + ½ (*M*_B1_*P*_B2_  *P*_B1_*M*_B2_) |
|  |  |  |
| *B*_1_*B*_2_ | *B*_1_*B*_1_ | ½ (*M*_B1_*M*_B1_ *P*_B2_*P*_B1_) + ½ (*M*_B1_*P*_B1_  *P*_B2_*M*_B1_) |
| *B*_1_*B*_2_ | *B*_1_*B*_2_ | ½ (*M*_B1_*M*_B1_ *P*_B2_*P*_B2_) + ½ (*M*_B1_*P*_B2_  *P*_B2_*M*_B1_) |
| *B*_1_*B*_2_ | *B*_2_*B*_1_ | ½ (*M*_B1_*M*_B2_ *P*_B2_*P*_B1_) + ½ (*M*_B1_*P*_B1_  *P*_B2_*M*_B2_) |
| *B*_1_*B*_2_ | *B*_2_*B*_2_ | ½ (*M*_B1_*M*_B2_ *P*_B2_*P*_B2_) + ½ (*M*_B1_*P*_B2_  *P*_B2_*M*_B2_) |
|  |  |  |
| *B*_2_*B*_1_ | *B*_1_*B*_1_ | ½ (*M*_B2_*M*_B1_ *P*_B1_*P*_B1_) + ½ (*M*_B2_*P*_B1_  *P*_B1_*M*_B1_) |
| *B*_2_*B*_1_ | *B*_1_*B*_2_ | ½ (*M*_B2_*M*_B1_ *P*_B1_*P*_B2_) + ½ (*M*_B2_*P*_B2_  *P*_B1_*M*_B1_) |
| *B*_2_*B*_1_ | *B*_2_*B*_1_ | ½ (*M*_B2_*M*_B2_ *P*_B1_*P*_B1_) + ½ (*M*_B2_*P*_B1_  *P*_B1_*M*_B2_) |
| *B*_2_*B*_1_ | *B*_2_*B*_2_ | ½ (*M*_B2_*M*_B2_ *P*_B1_*P*_B2_) + ½ (*M*_B2_*P*_B2_  *P*_B1_*M*_B2_) |
|  |  |  |
| *B*_2_*B*_2_ | *B*_1_*B*_1_ | ½ (*M*_B2_*M*_B1_ *P*_B2_*P*_B1_) + ½ (*M*_B2_*P*_B1_  *P*_B2_*M*_B1_) |
| *B*_2_*B*_2_ | *B*_1_*B*_2_ | ½ (*M*_B2_*M*_B1_ *P*_B2_*P*_B2_) + ½ (*M*_B2_*P*_B2_  *P*_B2_*M*_B1_) |
| *B*_2_*B*_2_ | *B*_2_*B*_1_ | ½ (*M*_B2_*M*_B2_ *P*_B2_*P*_B1_) + ½ (*M*_B2_*P*_B1_  *P*_B2_*M*_B2_) |
| *B*_2_*B*_2_ | *B*_2_*B*_2_ | ½ (*M*_B2_*M*_B2_ *P*_B2_*P*_B2_) + ½ (*M*_B2_*P*_B2_  *P*_B2_*M*_B2_) |

1. Frequencies of social interactions between genotype combinations at the C locus. The frequency values in the last column define the interaction frequencies vector for the C locus, **F_C_**.

| **Focal individual genotype** | **Social partner genotype** | **Frequency**  **(F_C_)** |
| --- | --- | --- |
| *C*_1_*C*_1_ | *C*_1_*C*_1_ | ½ (*M*_C1_*M*_C1_ *P*_C1_*P*_C1_) + ½ (*M*_C1_*P*_C1_  *P*_C1_*M*_C1_) |
| *C*_1_*C*_1_ | *C*_1_*C*_2_ | ½ (*M*_C1_*M*_C1_ *P*_C1_*P*_C2_) + ½ (*M*_C1_*P*_C2_  *P*_C1_*M*_C1_) |
| *C*_1_*C*_1_ | *C*_2_*C*_1_ | ½ (*M*_C1_*M*_C2_ *P*_C1_*P*_C1_) + ½ (*M*_C1_*P*_C1_  *P*_C1_*M*_C2_) |
| *C*_1_*C*_1_ | *C*_2_*C*_2_ | ½ (*M*_C1_*M*_C2_ *P*_C1_*P*_C2_) + ½ (*M*_C1_*P*_C2_  *P*_C1_*M*_C2_) |
|  |  |  |
| *C*_1_*C*_2_ | *C*_1_*C*_1_ | ½ (*M*_C1_*M*_C1_ *P*_C2_*P*_C1_) + ½ (*M*_C1_*P*_C1_  *P*_C2_*M*_C1_) |
| *C*_1_*C*_2_ | *C*_1_*C*_2_ | ½ (*M*_C1_*M*_C1_ *P*_C2_*P*_C2_) + ½ (*M*_C1_*P*_C2_  *P*_C2_*M*_C1_) |
| *C*_1_*C*_2_ | *C*_2_*C*_1_ | ½ (*M*_C1_*M*_C2_ *P*_C2_*P*_C1_) + ½ (*M*_C1_*P*_C1_  *P*_C2_*M*_C2_) |
| *C*_1_*C*_2_ | *C*_2_*C*_2_ | ½ (*M*_C1_*M*_C2_ *P*_C2_*P*_C2_) + ½ (*M*_C1_*P*_C2_  *P*_C2_*M*_C2_) |
|  |  |  |
| *C*_2_*C*_1_ | *C*_1_*C*_1_ | ½ (*M*_C2_*M*_C1_ *P*_C1_*P*_C1_) + ½ (*M*_C2_*P*_C1_  *P*_C1_*M*_C1_) |
| *C*_2_*C*_1_ | *C*_1_*C*_2_ | ½ (*M*_C2_*M*_C1_ *P*_C1_*P*_C2_) + ½ (*M*_C2_*P*_C2_  *P*_C1_*M*_C1_) |
| *C*_2_*C*_1_ | *C*_2_*C*_1_ | ½ (*M*_C2_*M*_C2_ *P*_C1_*P*_C1_) + ½ (*M*_C2_*P*_C1_  *P*_C1_*M*_C2_) |
| *C*_2_*C*_1_ | *C*_2_*C*_2_ | ½ (*M*_C2_*M*_C2_ *P*_C1_*P*_C2_) + ½ (*M*_C2_*P*_C2_  *P*_C1_*M*_C2_) |
|  |  |  |
| *C*_2_*C*_2_ | *C*_1_*C*_1_ | ½ (*M*_C2_*M*_C1_ *P*_C2_*P*_C1_) + ½ (*M*_C2_*P*_C1_  *P*_C2_*M*_C1_) |
| *C*_2_*C*_2_ | *C*_1_*C*_2_ | ½ (*M*_C2_*M*_C1_ *P*_C2_*P*_C2_) + ½ (*M*_C2_*P*_C2_  *P*_C2_*M*_C1_) |
| *C*_2_*C*_2_ | *C*_2_*C*_1_ | ½ (*M*_C2_*M*_C2_ *P*_C2_*P*_C1_) + ½ (*M*_C2_*P*_C1_  *P*_C2_*M*_C2_) |
| *C*_2_*C*_2_ | *C*_2_*C*_2_ | ½ (*M*_C2_*M*_C2_ *P*_C2_*P*_C2_) + ½ (*M*_C2_*P*_C2_  *P*_C2_*M*_C2_) |

**References:**

AUCLAIR, Y., KÖNIG, B., FERRARI, M., PERONY, N. & LINDHOLM, A. K. 2014. Nest attendance of lactating females in a wild house mouse population: benefits associated with communal nesting. *Animal Behavior,* 92**,** 143 - 149.

CHARALAMBOUS, M., COWLEY, M., GEOGHEGAN, F., SMITH, F. M., RADFORD, E. J., MARLOW, B. P., GRAHAM, C. F., HURST, L. D. & WARD, A. 2010. Maternally-inherited Grb10 reduces placental size and efficiency. *Developmental Biology,* 1**,** 1-8.

CHARALAMBOUS, M., SMITH, F. M., BENNETT, W. R., CREW, T. E., MACKENZIE, F. & WARD, A. 2003. Disruption of the imprinted *Grb10* gene leads to disproportionate overgrowth by an *Igf2*-independent mechanism. *Proceedings of the National Academy of Science USA,* 100**,** 8292-8297.

COWLEY, M., GARFIELD, A. S., MADON-SIMON, M., CHARALAMBOUS, M., CLARKSON, R. W., SMALLEY, M. J., KENDRICK, H., ISLES, A. R., PARRY, A. J., CARNEY, S., OAKEY, R. J., HEISLER, L. K., MOORWOOD, K., WOLF, J. B. & WARD, A. 2014. Developmental programming mediated by complementary roles of imprinted *Grb10* in mother and pup. *PLoS Biol,* 12**,** e1001799.

EBENSPERGER, L. A., HURTADO, M. J., SOTO-GAMBOA, M., LACEY, E. A. & CHANG, A. T. 2004. Communal nesting and kinship in degus (*Octodon degus*). *Naturwissenschaften,* 91**,** 391 - 395.

GARFIELD, A. S., COWLEY, M., SMITH, F. M., MOORWOOD, K., STEWART-COX, J. E., GILROY, K., BAKER, S., XIA, J., DALLEY, J. W., HURST, L. D., WILKINSON, L. S., ISLES, A. R. & WARD, A. 2011. Distinct physiological and behavioural functions for parental alleles of imprinted *Grb10*. *Nature,* 469**,** 534-538.

HAYES, L. D. 2000. To nest communally or not to nest communally: a review of rodent communal nesting and nursing. *Animal Behaviour,* 59**,** 677 - 688.

HAYES, L. D. & SOLOMON, N. G. 2004. Costs and benefits of communal rearing to female prairie voles (*Microtus ochrogaster*). *Behavioural Ecology and Sociobiology,* 56**,** 585-593.

KÖNIG, B. 1989. Kin Recognition and Maternal Care under Restricted Feeding in House Mice (*Mus domesticus*). *Ethology,* 82**,** 328-343.

KÖNIG, B. 1994a. Components of lifetime reproductive success in communally and solitarily nursing house mice -a laboratory study. *Behavioural Ecology and Sociobiology,* 34**,** 275 - 283.

KÖNIG, B. 1994b. Fitness effects of communal rearing in house mice: the role of relatedness versus familiarity. *Animal Behaviour,* 48**,** 1449 - 1457.

MACLEOD, K. J. & LUKAS, D. 2014. Revisiting non-offspring nursing: allonursing evolves when the costs are low. *Biology Letters,* 10**,** 20140378.

MANNING, C. J., DEWSBURY, D. A., WAKELAND, E. K. & POTTS, W. K. 1995. Communal nesting and communal nursing in house mice, *Mus musculus domesticus*. *Animal Behavior,* 50**,** 741-751.

PACKER, C., LEWIS, S. & PUSEY, A. 1992. A comparative analysis of non-offspring nursing. *Animal Behavior,* 43**,** 265 - 281.

PRICE, G. 1970. Selection and covariance. *Nature,* 227**,** 520-521.

RENFREE, M. B., SUZUKI, S. & KANEKO-ISHINO, T. 2013. The origin and evolution of genomic imprinting and viviparity in mammals. *Philos Trans R Soc Lond B Biol Sci,* 368**,** 20120151.

STRINGER, J. M., PASK, A. J., SHAW, G. & RENFREE, M. B. 2014. Post-natal imprinting: evidence from marsupials. *Heredity,* 113**,** 145 - 155.

STRINGER, J. M., SUZUKI, S., PASK, A. J., SHAW, G. & RENFREE, M. 2012. Selected imprinting of *INS* in the marsupial. *Epigenetics and Chromatin,* 5.

TYCKO, B. & MORISON, I. M. 2002. Physiological functions of imprinted genes. *Journal of Cellular Physiology,* 192**,** 245-258.

UBEDA, F. & GARDNER, A. 2015. Mother and offspring in conflict: why not? *PLOS Biology,* 13**,** e1002084.

ÚBEDA, F. & GARDNER, A. 2010. A model for genomic imprinting in the social brain: juveniles. *Evolution,* 64**,** 2587 - 2600.

ÚBEDA, F. & GARDNER, A. 2011a. A model for genomic imprinting in the social brain: adults. *Evolution,* 65**,** 462 - 475.

ÚBEDA, F. & GARDNER, A. 2011b. A model for genomic imprinting in the social brain: elders. *Evolution,* 66**,** 1567 - 1581.

WOLF, J. B. 2013. The evolution of genomic imprinting as a coordinator of coadapted gene expression. *Proceedings of the National Academy of Sciences, USA,* 110**,** 5085-5090.

WOLF, J. B., COWLEY, M. & WARD, A. 2015. Coadaptation between mother and offspring: why not? *PLOS Biology,* 13**,** e1002085.

WOLF, J. B. & HAGER, R. 2006. A maternal-offspring coadaptation theory for the evolution of genomic imprinting. *PLoS Biology,* 4**,** e380.
